# Long-duration aqueous manganese metal anode by hydrogel solvation regulation

**DOI:** 10.1126/sciadv.aeg1770

**Published:** 2026-09-25

**Authors:** Hongbao Zheng, Xinyuan Zhang, Wenqiang Lu, Zhichao Hou, Zhixuan Wei, Nan Chen, Dong Zhang, Heng Jiang, Fei Du

**Affiliations:** ^1^ Key Laboratory of Physics and Technology for Advanced Batteries (Ministry of Education), State Key Laboratory of Superhard Materials, College of Physics, Jilin University, Changchun 130012, China.; ^2^ School of Physical and Mathematical Sciences, Nanyang Technological University, Singapore 637371, Singapore.; ^3^ Zhengzhou Research Institute, Harbin Institute of Technology, Zhengzhou 450000, P. R. China.

## Abstract

The key challenge of implementing aqueous manganese (Mn) metal batteries is severe water-related parasitic reactions, including the hydrogen evolution reaction and Mn corrosion. These issues originate largely from the high reactivity of solvated water around manganese ions (Mn^2+^). Herein, we design a hydrogel electrolyte that reconstitutes the Mn^2+^ solvation structure and establishes a hydration-regulated ion-migration environment. The incorporated 18-crown-6 macrocycles on polymer chains bind strongly to Mn^2+^, reducing its hydration number from 5.51 to 1.39 and forming a water-repelling, polymer-guided conduction pathway. Dynamic measurements demonstrate uniform Mn deposition and suppressed detrimental gas evolution. Consequently, Mn plating/stripping in this hydrogel becomes highly reversible, achieving an average Coulombic efficiency of 95% over 350 cycles (Mn||Cu cells) and low polarization of ∼24 millivolts for over 1800 hours (Mn||Mn cells). Moreover, pouch cells paired with a silver vanadium oxide (AgVO) cathode (N/P ratio of 4.63) retain 95.3% of the initial capacities after 200 cycles. This work demonstrates an effective solvation-regulation strategy via hydrogel design for durable Mn anodes in aqueous batteries.

## INTRODUCTION

**Fig. 1.**
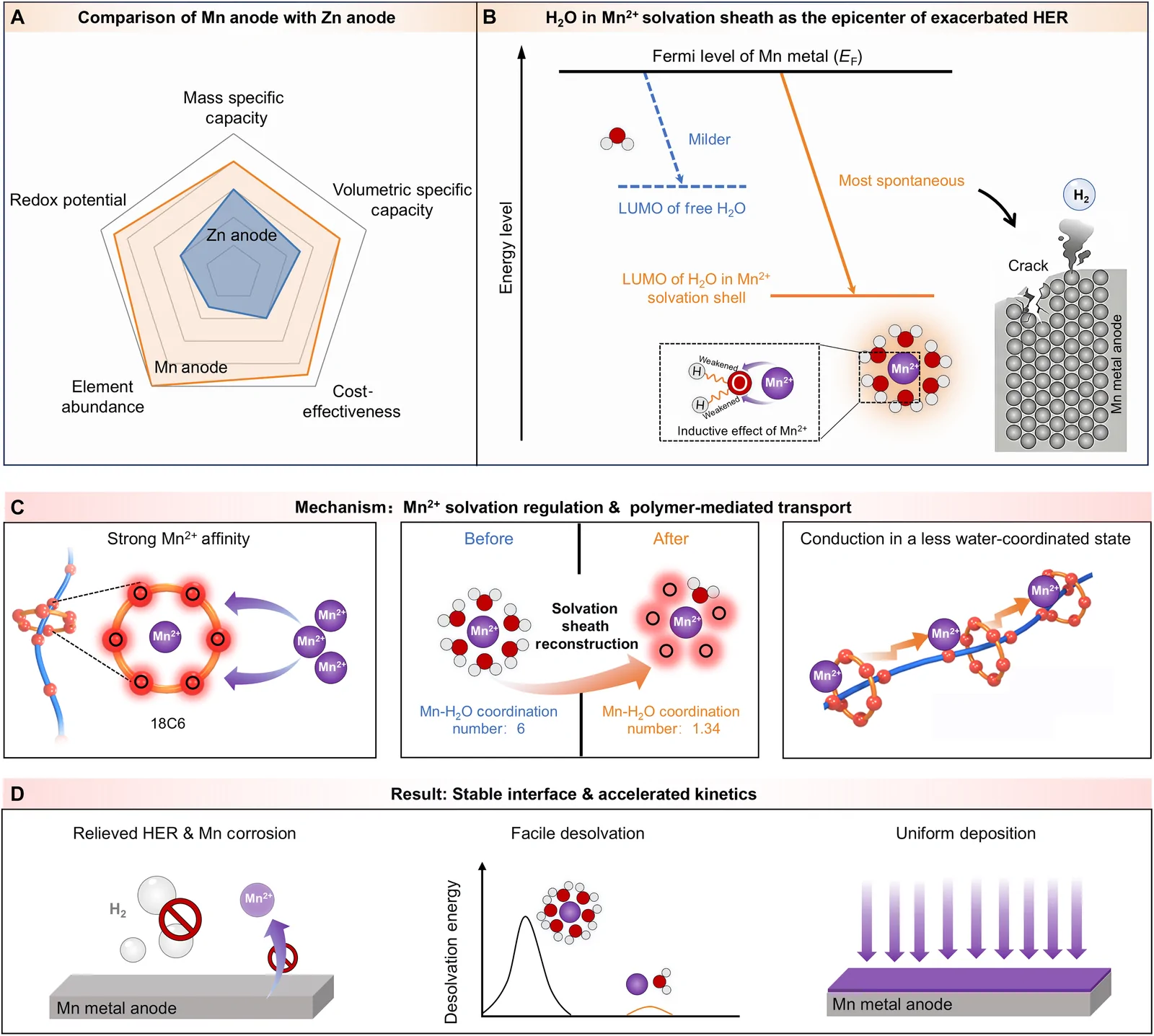
Design framework for realizing the long-duration aqueous Mn metal anode. (**A**) Comparison of Mn and traditional Zn metal anodes (see details in table S1). (**B**) Analysis of HER origins. Water molecules in the Mn^2+^ solvation shell are the primary cause of the HER in AMMBs. (**C** and **D**) Schematic illustration of the function of PPR/PAM in regulating the solvation structure and migration environment of Mn^2+^, which renders robust and fast Mn plating/stripping.

Aqueous metal batteries have emerged as a promising, safe, cost-effective, and environmentally benign technology for large-scale energy storage (*1*–*3*). Manganese (Mn) metal anodes have the advantages of abundant Earth crust’s reserves (ranking 12th), mature processing technology, and high theoretical capacity (976 mA·hour g^−1^) with low redox potential [−1.19 V versus standard hydrogen electrode (SHE)] (Fig. 1A and table S1) (*4*–*6*). Nevertheless, its redox potential comes with intrinsic challenges, which reside far below that of the notorious hydrogen evolution reaction (HER). Moreover, the issue is intensified by Mn’s electronic configuration, where the high-energy unpaired d electrons can function as a potent catalyst for the HER. These factors simultaneously trigger severe metal corrosion, accompanied by insoluble and inert by-product formation, which inevitably reduces the Mn plating/stripping Coulombic efficiency (CE) and poses safety risks in aqueous Mn metal batteries (AMMBs). As a result, the currently reported AMMBs suffer from poor Mn metal utilization (CE < 75%) (see details in table S2) (*7*–*10*), representing a critical hurdle to achieving long-term cycling stability and practical implementation.

The fundamental key to suppressing the HER lies in reducing the activity of water molecules that are in direct contact with the metal anode surface (*11*). The current study on a similar Zn anode system shows that hydrogel electrolytes can effectively mitigate water-related side reactions with CE well exceeding 95% (*12*–*14*). However, in our previous attempt, a cross-linked sodium alginate@Mn hydrogel could not achieve a CE exceeding 60% for Mn plating/stripping in AMMBs (*8*). This indicates the intrinsic difference in HER mechanisms between the Zn and Mn anodes. Previous studies have indicated that the cation-water interaction can polarize the O─H bonds, rendering them more susceptible to dissociation (Fig. 1B) (*15*–*17*). Therefore, the lowest unoccupied molecular orbital (LUMO) energy of water is lowered in its bound state so that the solvated water is more prone to reduction than the free water. In case of Mn, this energy disparity will be more obvious owing to the inductive effect of Mn^2+^ ions, thereby leading to an even higher proportion of HER contribution by the solvated H_2_O molecules. This likely explains the limited effectiveness of common hydrogel electrolytes in AMMBs, which predominantly focus on restricting bulk free water and consider less the more reactive solvated species.

This insight underscores the necessity of minimizing water participation in the Mn^2+^ solvation shell. However, a fundamental trade-off persists: Water molecules are indispensable in facilitating Mn^2+^ transport. Conventional strategies, such as water-in-salt electrolytes (*18*, *19*) or high-donor-number organic solvents (*20*, *21*), aim to reduce solvated water but often compromise cost-effectiveness or ion conductivity. Therefore, a critical research gap remains in designing holistic electrolyte systems that concurrently modulate cation-water-polymer interactions to optimize solvation structure, ion transport, and interfacial stability. Our intention is to fill this gap by rationally designing the hydrogel electrolyte, aiming for long-term cycling stability and high redox reversibility in AMMBs.

Herein, our hydrogel electrolyte is constructed via cross-linking pseudo-polyrotaxane (PPR) and polyacrylamide (PAM). Self-assembled from polyethylene glycol (PEG) and 18-crown-6 (18C6), the PPR framework exhibits concentrated negative charges in 18C6 cavities that selectively capture Mn^2+^ ions (Fig. 1C). This interaction effectively restructures the Mn^2+^ solvation sheath, reducing the Mn-H_2_O coordination number (CN) to 1.39. More notably, PPR/PAM establishes a polymer-dominated ion transport pathway that prevents the formation of highly hydrated Mn^2+^ species during migration. The modified solvation environment not only alleviates HER and Mn corrosion but also facilitates interfacial desolvation, thereby stabilizing and accelerating Mn deposition (Fig. 1D). Consequently, the Mn||Cu asymmetric cells achieve highly reversible Mn plating/stripping with a state-of-the-art average CE of 95% over 350 cycles. The Mn||Mn symmetric cells maintain a stable overpotential of ∼24 mV over 1800 hours at 0.1 mA cm^−2^. Furthermore, a foldable pouch cell configured with a AgVO cathode delivers 251 mA·hour g^−1^ at 0.5 A g^−1^ and retains 95.3% of its capacity after 200 cycles. This work provides valuable insights into the rational design of hydrogel electrolytes for highly reversible and durable AMMBs.

## RESULTS

### Design and construction of the PPR/PAM hydrogel

**Fig. 2.**
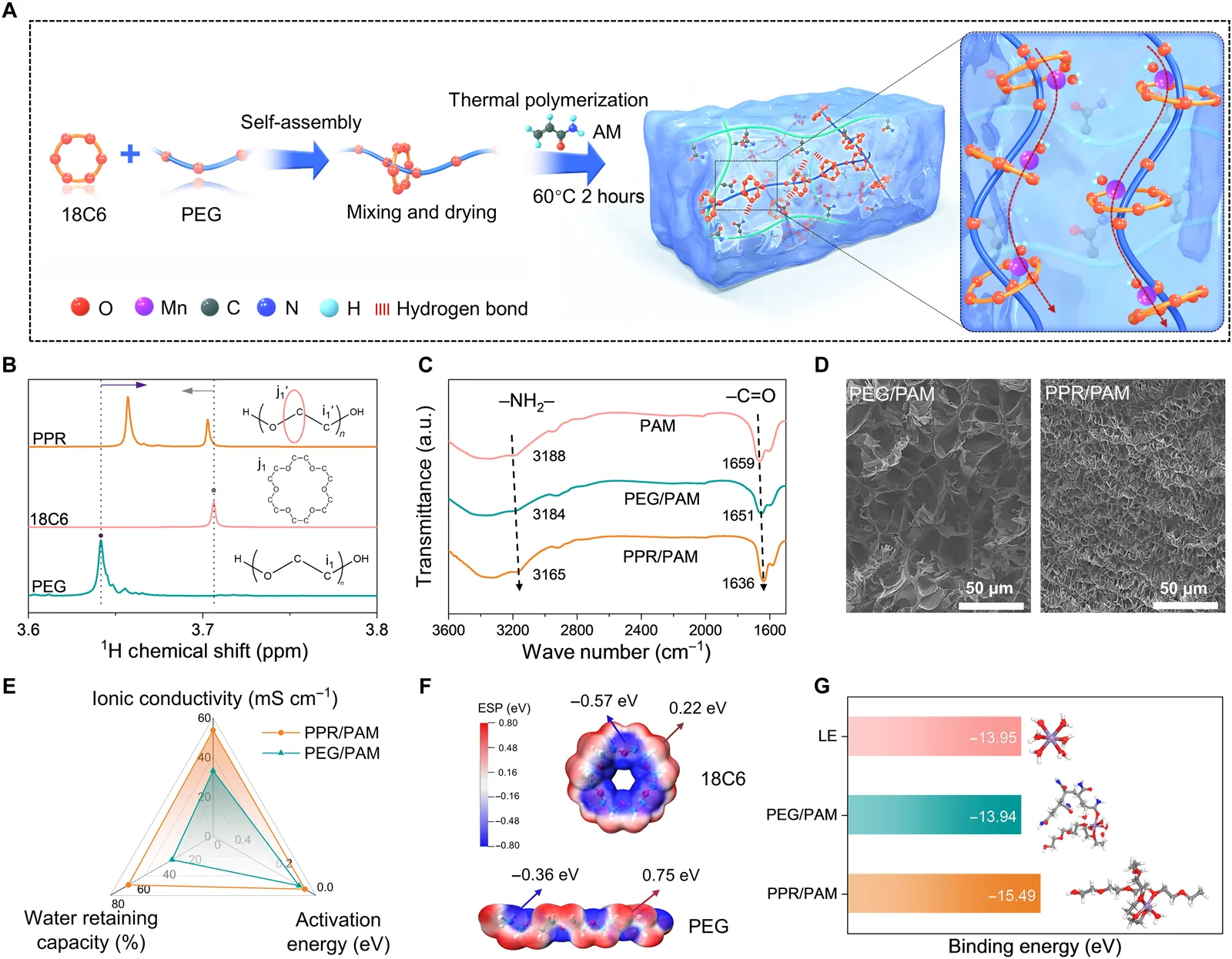
Synthesis route and characterization of the targeted hydrogel. (**A**) Schematic illustration for the synthesis and microstructure of the PPR/PAM hydrogel electrolyte. (**B**) Solid-state ^1^H MAS NMR spectra of PEG, 18C6, and PPR, together with resonance assignments. ppm, parts per million. (**C**) FTIR spectra of PAM, PEG/PAM, and PPR/PAM. a.u., arbitrary units. (**D**) Cross-sectional SEM images of freeze-dried PEG/PAM and PPR/PAM. (**E**) Comparison radar chart of key performances of PPR/PAM and PEG/PAM. (**F**) ESP mapping of 18C6 and PEG. (**G**) Binding energy comparison of Mn^2+^-O in different electrolyte systems, where the representative solvation structure is obtained by MD simulation.

Crown ethers are structurally simple macrocycles renowned for their ability to selectively coordinate various metal cations (*22*, *23*). Capitalizing on this unique characteristic, a previously unknown hydrogel electrolyte, PPR/PAM, was designed to regulate Mn^2+^ coordination and stabilize the Mn metal anode. The selection of 18C6 and manganese perchlorate salt was guided by a comprehensive investigation of their electrochemical performance (fig. S1). The PPR/PAM was synthesized via a step-growth polymerization process, as illustrated in Fig. 2A (see Materials and Methods for detailed information). The successful formation of the desired PPR complex and PPR/PAM interpenetrating network was validated by multiple characterization techniques. Proton nuclear magnetic resonance (^1^H NMR) reveals sharp signals at 3.70 and 3.64 parts per million (ppm), assigned to the ─OCH_2_CH_2_─ protons of 18C6 and PEG, respectively (Fig. 2B) (*24*, *25*). Upon complexation, the PEG proton signal experiences a downfield shift due to the deshielding effect within the crown ether cavity, whereas the 18C6 proton signal shifts upfield, representing the increased electron density (*26*, *27*). Fourier transform infrared spectroscopy (FTIR) also corroborates the interactions between PEG and 18C6 (fig. S2) (*28*, *29*). In addition, the second-step cross-linking between PPR and PAM is confirmed by FTIR analysis. The ─NH_2_─ and C═O stretching vibrations red shift, attributable to physical entanglement and hydrogen bonding between the two polymer chains (Fig. 2C) (*30*). For comparison, the PEG/PAM hydrogel (without 18C6) displays intermediate peak positions in the FTIR spectra, suggesting the critical role of 18C6 macrocycles in reinforcing cross-linking efficiency.

The ionic conductivity of PPR/PAM was optimized to 53.8 mS cm^−1^ by tuning the ratio of PPR, PAM, and Mn salt (fig. S3). The value is lower than that of the aqueous liquid electrolyte [1 M Mn(ClO_4_)_2_, denoted as LE] (98.0 mS cm^−1^) but exceeds that of PEG/PAM (33.4 mS cm^−1^; fig. S4 and table S3). Therefore, the rational hydrogel design has facilitated the ion transport, accompanied by a lowered activation energy for Mn^2+^ conduction, 0.052 eV for PPR/PAM compared with 0.081 eV for PEG/PAM (fig. S5). Scanning electron microscopy (SEM) images of the freeze-dried hydrogels reveal sharp morphological differences (Fig. 2D and fig. S6). The PPR/PAM hydrogel exhibits substantially smaller pore sizes (1 to 4 μm) compared to PEG/PAM (12 to 23 μm), reflecting a compact microstructure with a denser cross-linked network arising from enhanced interchain interactions. Under ambient conditions (25°C, 30% relative humidity), PPR/PAM retains 66% of its initial water content after 50 hours, notably outperforming PEG/PAM (32% retention; fig. S7). The above benefits establish PPR/PAM as a comprehensively enhanced hydrogel system in key metrics, including ionic transport and water retention (Fig. 2E).

The Mn^2+^ affinity of the designed hydrogel was revealed by density functional theory (DFT) calculations. The electrostatic potential (ESP) mapping (Fig. 2F) shows that all oxygen atoms of 18C6 are oriented toward the inner cavity, generating a highly concentrated region of negative charge density (up to −0.57 eV). Bare PEG chains display alternating positive and negative charge distributions with a lower negative charge density (maximum −0.36 eV). The negative charge localized within the 18C6 cavities of the PPR framework induced strong electrostatic interactions with Mn^2+^, leading to a binding energy of −15.49 eV for the six-coordinate configuration (including one H_2_O). This value is more negative than the calculated binding energies of −13.94 eV for the Mn^2+^─[H_2_O]_6_ and − 13.95 eV for Mn^2+^ with PEG/PAM, thereby highlighting the preferential confinement mechanism of the PPR structure (Fig. 2G and fig. S8).

### Dynamic solvation structure regulation

**Fig. 3.**
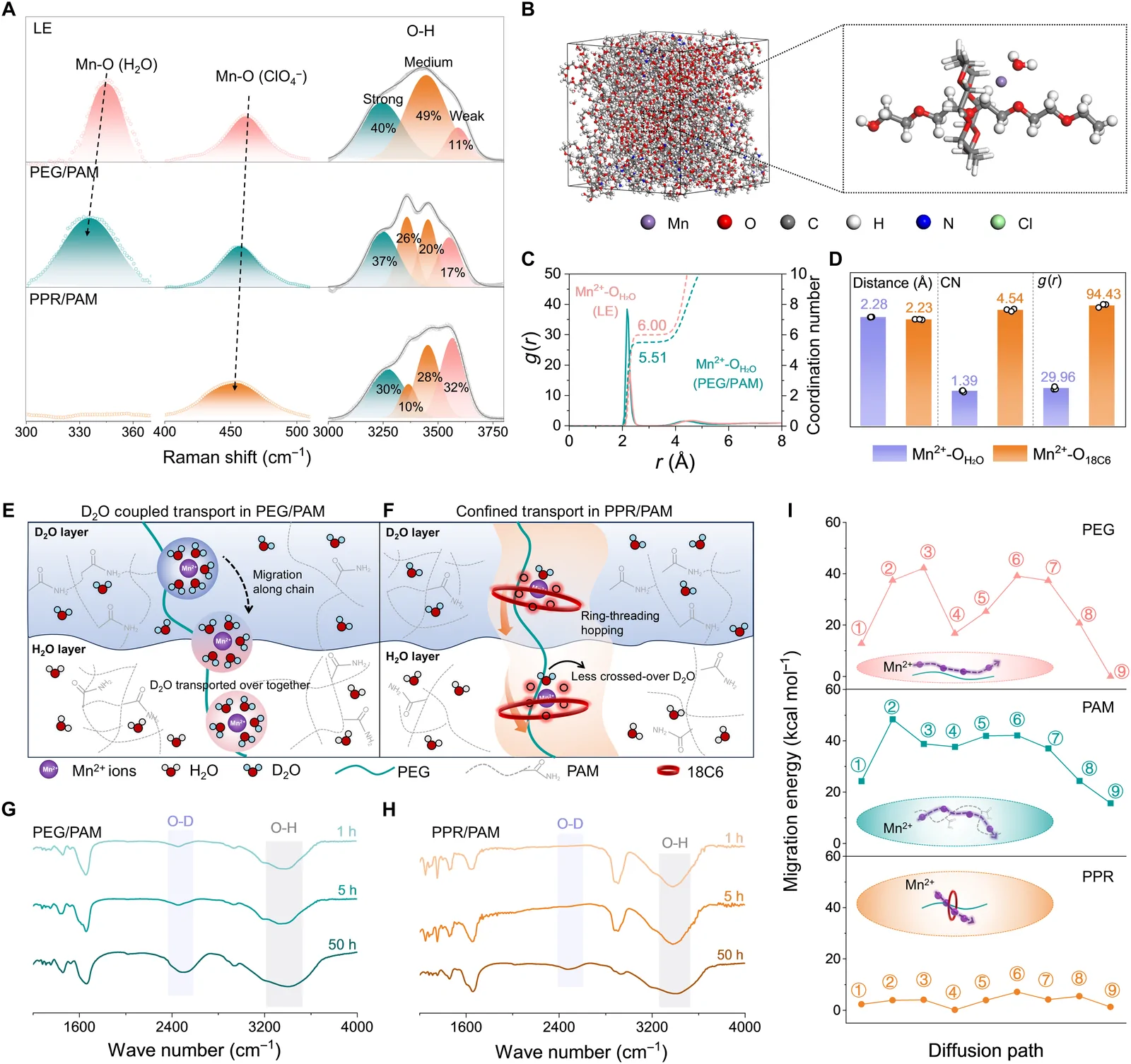
Solvation structure analysis of hydrogel electrolytes. (**A**) Raman spectra of different electrolytes. (**B**) 3D snapshots of the PPR/PAM system and the enlarged image of the representative solvation structure obtained from the MD simulation. (**C**) RDFs and integral curves of Mn^2+^-O_H2O_ in LE and PEG/PAM. (**D**) Distance, CN, and *g*(*r*) values of Mn^2+^-O_H2O_ and Mn^2+^-O_18C6_ in PPR/PAM. Schematic diagram of isotope calibration experiments and ion transport mechanisms in (**E**) PEG/PAM and (**F**) PPR/PAM. FTIR spectra of the upper layer of the H_2_O-based electrolyte in (**G**) PEG/PAM and (**H**) PPR/PAM systems. h, hours. (**I**) Comparison of Mn^2+^ migration energy along with the transport pathway for PEG, PAM, and PPR (see more details in fig. S16).

The detailed intermolecular interactions in various electrolyte systems were investigated. Figure 3A presents Raman spectra of the three comparative systems. In LE, all Mn ions are trapped in the water network, whose strong coordination leads to a characteristic Mn─O symmetric stretching vibration at 357 cm^−1^ (*31*). Notably, this peak exhibits progressive red shift and broadening in PEG/PAM and eventually becomes nearly undetectable in PPR/PAM, illustrating a systematic disruption of the Mn^2+^-H_2_O coordination (*32*). A similar trend is observed in the ν(ClO_4_^−^) vibration, indicating weakened electrostatic interactions between Mn^2+^ and ClO_4_^−^ anions (*33*). Furthermore, the C-O-C stretching mode in PPR/PAM displays a blue shift compared to the manganese-free hydrogel (fig. S9), providing direct evidence for Mn^2+^ coordination with 18C6 macrocycles (*34*). These spectroscopic observations, in agreement with DFT predictions, indicate that Mn^2+^ of PPR/PAM is confined within the 18C6 cavities, simultaneously stripping multiple H_2_O molecules from the Mn^2+^-primary solvation shells. In addition, the hydrogel matrix modifies the hydrogen-bond network of water. Upon introducing 18C6 into the hydrogel, the proportion of strong hydrogen bonds decreases whereas that of weak hydrogen bonds increases, further evidenced by the O─H bond strength analysis via vibrational density of states (VDOS) (Fig. 3A and fig. S10) (*35*–*37*). This shift is attributed to the densely packed polymer chains, which disrupt native H_2_O-H_2_O interactions via establishing a framework richer in H-bond donors and acceptors (*38*). Consistent with these spectroscopic results, molecular dynamics (MD) simulations further reveal that PPR/PAM (three trails) has a more disrupted hydrogen-bond network and a slightly longer average H-bond distance than PEG/PAM, directly reflecting polymer-induced water restructuring (fig. S11 and table S4) (*37*).

MD simulations further reveal the microscopic characteristics of the different electrolyte systems (table S5). Analysis of the radial distribution functions (RDFs) and CNs confirms the classic [Mn(H_2_O)_6_]^2+^ configuration in the LE system (Fig. 3C and fig. S12A). In the PEG/PAM hydrogel, the CN of Mn─O (H_2_O) decreases slightly to 5.51, with PAM chains partially participating in the primary solvation shell of Mn^2+^ (Fig. 3C and fig. S12B). More notably, the simulation of the PPR/PAM system shows that Mn^2+^ is localized within the cavities of 18C6 (Fig. 3B). The average Mn─O (18C6) bond length is 2.23 Å, closely matching the average Mn─O (H_2_O) distance of 2.28 Å, which demonstrates that 18C6 is successfully incorporated into the primary solvation shell of Mn^2+^. Concomitantly, the average Mn─H_2_O CN drops markedly to 1.39. The corresponding peak height of *g*(*r*) for Mn─O (18C6) (94.43) far exceeds that for Mn─O (H_2_O) (29.96), further underscoring the strong affinity of Mn^2+^ for the crown ether ligand (Fig. 3D and fig. S13).

In addition to the static solvation structure, the dynamic evolution of solvation during electrochemical ion transfer is also critical. To elucidate this dynamic behavior, an isotope-labeling strategy was used. Upon charging, Mn ions undergo directional migration from the D_2_O to H_2_O layer. Time-dependent FTIR analysis shows a pronounced intensification of the O-D vibrational signature in PEG/PAM, corresponding to transport of highly hydrated Mn ions across the D_2_O/H_2_O interface (Fig. 3, E and G) (*39*). In striking contrast, PPR/PAM displays a marginal increase in the characteristic O-D peak (Fig. 3H), implying that the PPR structure has a water-repelling property throughout the transport process, corroborated by ex situ Raman spectra (fig. S14). Static control experiments (zero applied potential) indicate that passive Fickian diffusion of D_2_O is negligible in PPR/PAM, further supporting that the O-D signal originates predominantly from electromigration with Mn ions (fig. S15). Given the intrinsic correlation between the ion-conduction mechanism and the evolving solvation environment, we infer that the Mn ions predominantly migrate along polymer-bound pathways rather than being released into the surrounding water medium. Moreover, in combination with MD simulation, Mn ions in PPR/PAM are likely to diffuse as [Mn(H_2_O)]^2+^ complexes to suppress water diffusion and enhance interfacial stability, as illustrated in Fig. 3F.

Theoretical calculations of Mn^2+^ migration pathways provide fundamental insights into the conduction mechanism. According to the calculated migration energies (Fig. 3I), transport along PPR chains occurs via a distinctive ring-threading mechanism between 18C6 cavities and adjacent PEG coordination sites (see the detailed migration pathway in fig. S16). The electronegative cavity of 18C6 facilitates selective Mn^2+^ permeation, whereas the combined effects of strong coordination and concentration gradients create an efficient ion-pumping effect. Such transport pathway exhibits an average migration barrier one order of magnitude lower than that along PEG and PAM chains. Combining Fig. 3G and Fig. 3I, we infer that the compact network in PPR/PAM (as shown by SEM images in Fig. 2E) will create continuous, tightly packed conduction channels along the polymer backbone.

Together, the molecular design of PPR/PAM not only tailors the Mn^2+^ solvation structure in the hydrogel environment but also enables their migration in a less water-coordinated state. Regarding the effects in AMMBs, we expect this hydrogel electrolyte to accelerate Mn deposition kinetics and reduce water-induced parasitic reactions (to be discussed below).

### Mn plating/stripping behavior

**Fig. 4.**
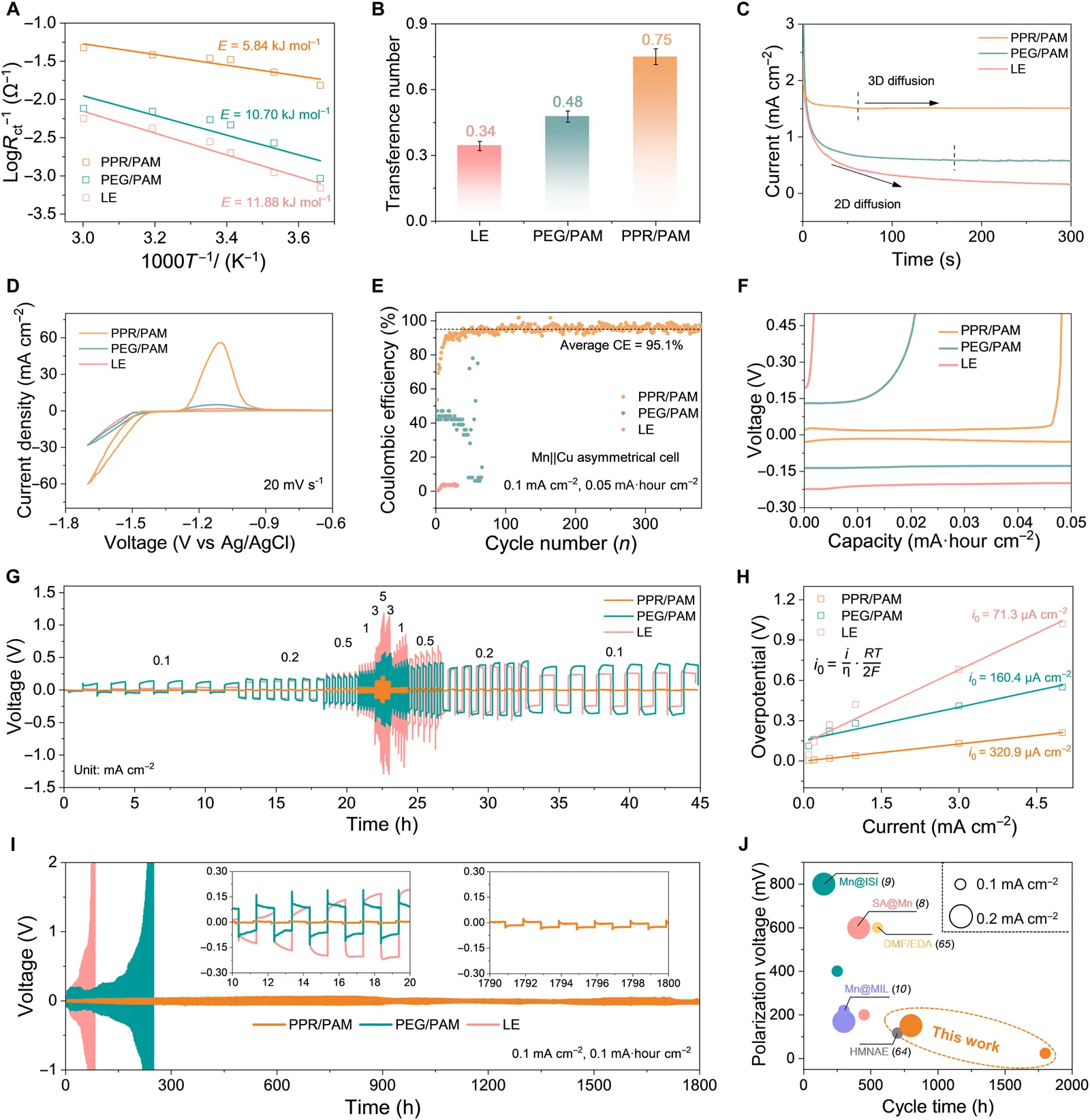
Mn plating/stripping behaviors in different electrolytes. (**A**) Arrhenius curves and corresponding activation energies on the Mn surface achieved with different electrolytes. (**B**) Mn^2+^ transference number of different electrolytes. (**C**) Chronoamperometric curves of symmetric cells in different electrolytes. (**D**) CV curves of Mn||Cu asymmetric cells in different electrolytes. (**E**) CEs and (**F**) plating/stripping curves of Mn||Cu half cells at 0.1 mA cm^−2^ and 0.05 mA·hour cm^−2^. (**G**) Rate performance and (**H**) exchange current densities of symmetric cells. (**I**) Cycling performance of Mn||Mn symmetric cells at 0.1 mA cm^−2^ and 0.1 mA·hour cm^−2^ (insets: magnified voltage profiles). (**J**) Symmetric cell cycling performance compared with reported Mn systems (*8*–*10*, *64*, *65*).

Electrochemical characterization of symmetric and asymmetric cells was performed to evaluate differences in Mn plating/stripping behavior induced by coordination chemistry in PPR/PAM. The Mn^2+^ activation energy of the symmetric cells with PPR/PAM electrolyte drops to 5.84 kJ mol^−1^, compared to 10.70 kJ mol^−1^ for PEG/PAM and 11.88 kJ mol^−1^ for LE (Fig. 4A and fig. S17). This difference should originate from the reduced number of coordinated H_2_O molecules, thereby effectively facilitating interfacial desolvation (*40*). The cation transference number (*t*^+^) further highlights the superiority of PPR/PAM, reaching 0.75 compared to 0.48 for PEG/PAM and 0.34 for LE (Fig. 4B and fig. S18), which is important for mitigating concentration polarization during cycling. Chronoamperometric analysis reveals that Mn deposition in PEG/PAM and LE follows a two-dimensional (2D) nucleation pathway (Fig. 4C). In contrast, in the PPR/PAM system, the fast mass transfer and desolvation drive a 3D diffusion-controlled growth, beneficial for the formation of a compact and homogeneous metallic Mn deposition morphology (*41*).

As shown in Fig. 4D, the cyclic voltammetry (CV) curve of the PPR/PAM-based Mn||Cu asymmetric cell presents a lower nucleation overpotential and distinct redox peaks, whereas a markedly weakened oxidation peak was observed for LE, likely due to the combined effects of H_2_O-induced parasitic reactions on the Mn surface and sluggish Mn plating/stripping kinetics. The electrochemical reversibility is quantified by CE measurements in Mn||Cu cells. Both PEG/PAM and LE systems display low CE and fail shortly within 50 cycles. In sharp contrast, the PPR/PAM system maintains stable cycling with an average CE value of 95.1% over 350 cycles (Fig. 4E). Moreover, multiple commercial current collectors also exhibit reversible Mn plating/stripping with high CEs, confirming that the improved CE originates from the PPR/PAM hydrogel rather than from the Cu current collector (fig. S19). Notably, at a higher current density of 1 mA cm^−2^ and a capacity of 0.2 mA·hour cm^−2^, the system achieves an even higher CE of 98.6% over 500 cycles (fig. S20 and table S6). In addition, it demonstrates the lowest voltage polarization among the three electrolytes (Fig. 4F). When the current density increases from 0.1 to 5 mA cm^−2^, the polarization voltage of the PPR/PAM-based Mn||Mn symmetric cell exhibits only a minimal increase of 0.18 V, and a negligible hysteresis is noted when it returns to the initial current density of 0.1 mA cm^−2^ (Fig. 4G). In stark contrast, cells using PEG/PAM or LE show poor rate tolerance, as reflected in pronounced voltage fluctuations and hysteresis. The exchange current of Mn||PPR/PAM||Mn (320.9 μA cm^−2^), more than double the values from PEG/PAM (160.4 μA cm^−2^) and LE (71.3 μA cm^−2^), also demonstrates the enhanced charge transfer kinetics (Fig. 4H).

The long-term compatibility between PPR/PAM and the Mn metal anode was further substantiated in symmetric cell tests. At a current density of 0.1 mA cm^−2^, Mn||PPR/PAM||Mn exhibits stable voltage separation at ∼23.6 mV over 1800 hours (Fig. 4I). At a doubled current density (0.2 mA cm^−2^), the Mn striping/plating can continue up to 800 hours (fig. S21). Conversely, cells using PEG/PAM or LE could only survive for less than 200 hours. Even under intermittent cycling conditions (aging between cycles), Mn||PPR/PAM||Mn can sustain over 250 hours (fig. S22). Compared with prior aqueous Mn||Mn symmetric cells (Fig. 4J and table S7), the PPR/PAM electrolyte delivers superior stripping/plating kinetics and cycling stability, making itself an enabling system for durable AMMBs.

### Effectiveness in interfacial stabilization

**Fig. 5.**
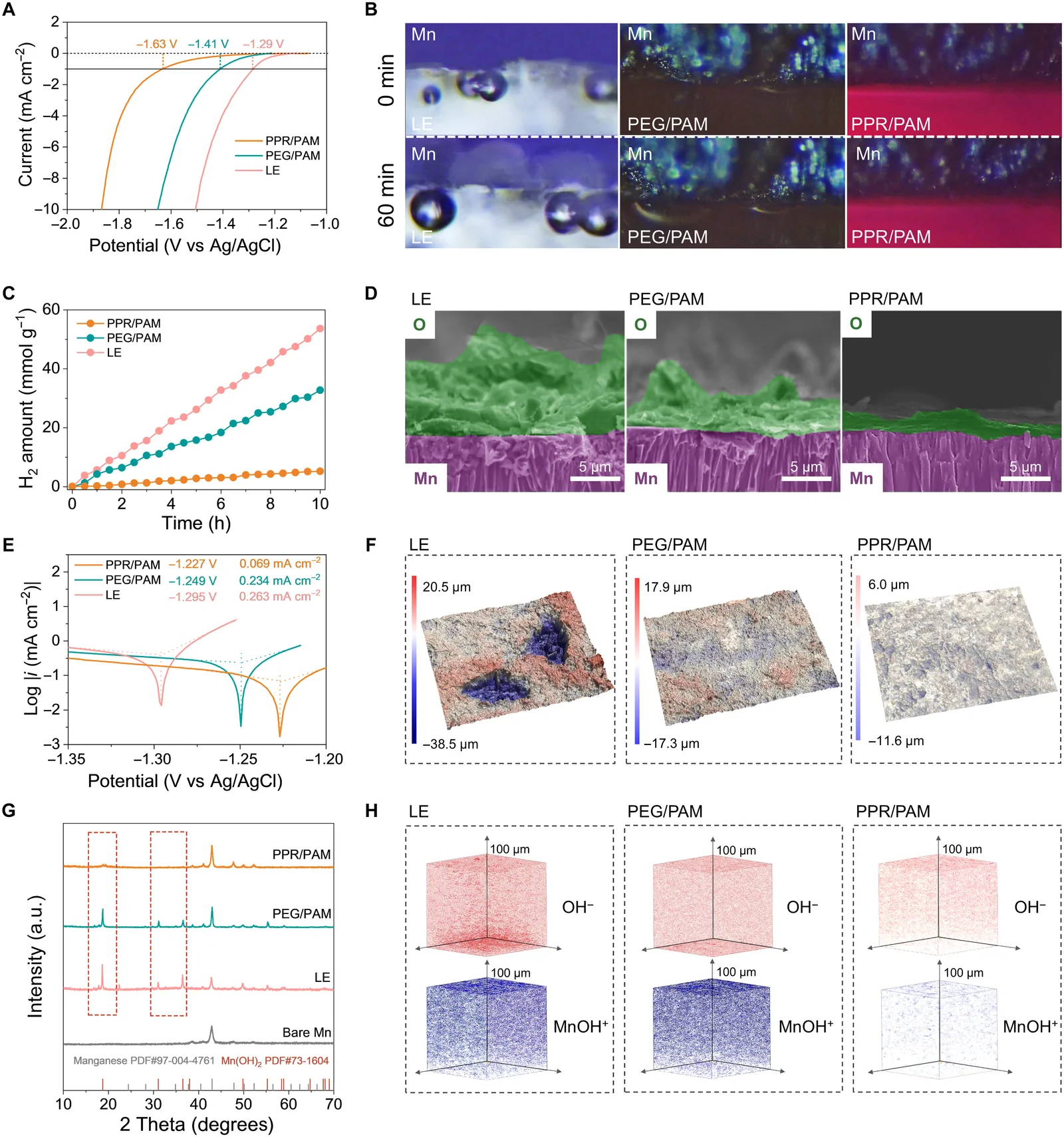
Characterizations of the interfaces. (**A**) LSV curves of the three electrolytes. (**B**) Optical microscopy images of Mn electrodes at 0 and 60 min deposition at 0.1 mA cm^−2^ in different electrolytes. (**C**) Variation curve of hydrogen production and voltage-time plots for the Mn||Mn symmetric cell at 0.1 mA cm^−2^. (**D**) False-colored SEM cross-sectional images of the Mn anode after 100 cycles in different electrolytes. (**E**) Linear polarization curves achieved with different electrolytes. (**F**) 3D laser optical images of the Mn anode surface cycled in different electrolytes. (**G**) XRD patterns of Mn anodes after 100-hour cycle at 0.1 mA cm^−2^ with different electrolytes. Dashed boxes indicate the position for side-reaction products. (**H**) TOF-SIMS 3D spectra of the Mn anode surface cycled after 100 times in different electrolytes.

During plating in conventional aqueous Mn^2+^ electrolytes, [Mn(H_2_O)_6_]^2+^ complexes migrate to the electrode surface, where the highly active solvated water molecules are prone to decompose, exacerbating the HER and triggering severe parasitic side reactions (*42*). In the PPR/PAM electrolyte, the dynamic solvation strategy ensures that Mn ions approach the electrode in a low-hydrated state, thereby establishing a critical barrier against HER. Moreover, the stronger O─H bonds and more disrupted hydrogen bond network in PPR/PAM further render the Volmer step of the HER more difficult, intrinsically lowering its HER activity. To verify the above perspective, systematic electrochemical and morphological experiments were conducted. First, linear sweep voltammetry (LSV) suggests that the PPR/PAM hydrogel could effectively shift the HER threshold from −1.29 V (versus Ag/AgCl) for LE to −1.63 V for PPR/PAM, thereby enabling a broader electrochemical stability window (ESW) of 2.4 V (Fig. 5A and fig. S23). Optical microscopy images, along with gas chromatography (GC) measurements, confirm the reduction in HER activity. LE and PEG/PAM yield bubble-covered and irregular Mn surfaces during electrodeposition, whereas PPR/PAM maintains a smooth electrode morphology with negligible gas evolution (Fig. 5B and fig. S24). The cumulative hydrogen evolution after 10 cycles in PPR/PAM accounts for only 16.1 and 9.8% of that observed in PEG/PAM and LE, respectively (Fig. 5C). The constrained gas generation is critical for achieving uniform metal deposition, eliminating current hotspots and preventing galvanic corrosion. As shown in Fig. 5E, the corrosion potential of Mn in PPR/PAM shifts positively to −1.28 V (versus Ag/AgCl), and the corrosion current density is reduced to 0.07 mA cm^−2^, as compared to 0.23 mA cm^−2^ in PEG/PAM and 0.26 mA cm^−2^ in LE, suggesting reduced tendency and rate of Mn corrosion.

Correspondingly, with LE and PEG/PAM electrolytes, by-products are accumulated after prolonged cycling. Randomly distributed oxygen-containing by-products and severe surface roughening are observed after 100 cycles (Fig. 5D and fig. S25). Conversely, PPR/PAM maintains a smooth Mn surface with minimal morphological deterioration. Quantitative 3D laser confocal microscopy confirms this difference. Among all systems, the PPR/PAM exhibits the lowest Sa (arithmetic mean height), Sdr (developed surface area ratio), and Spc (arithmetic mean peak curvature), along with a Str (texture aspect ratio) approaching unity, thereby demonstrating a smooth, isotropic, and dendrite-less Mn deposition (Fig. 5F and table S8). The postcycle x-ray diffraction (XRD) analysis reveals the formation of Mn(OH)_2_ on the Mn metal in LE and PEG/PAM, which is nearly absent in the PPR/PAM system (Fig. 5G). Consistent with XRD observations, x-ray photoelectron spectroscopy (XPS) analysis shows a pronounced shift of the Mn 2p peak toward higher binding energies in LE and PEG/PAM systems (fig. S26), corresponding to substantial surface Mn^2+^ formation (*43*). Last, in the time-of-flight secondary ion mass spectrometry (TOF-SIMS) depth profiles, OH^−^ and MnOH^+^ fragments exhibit abundant distributions in control electrolytes, whereas these corrosion by-products are only sparsely distributed in PPR/PAM (Fig. 5H and fig. S27).

### Quasi-solid-state Mn metal full cells

**Fig. 6.**
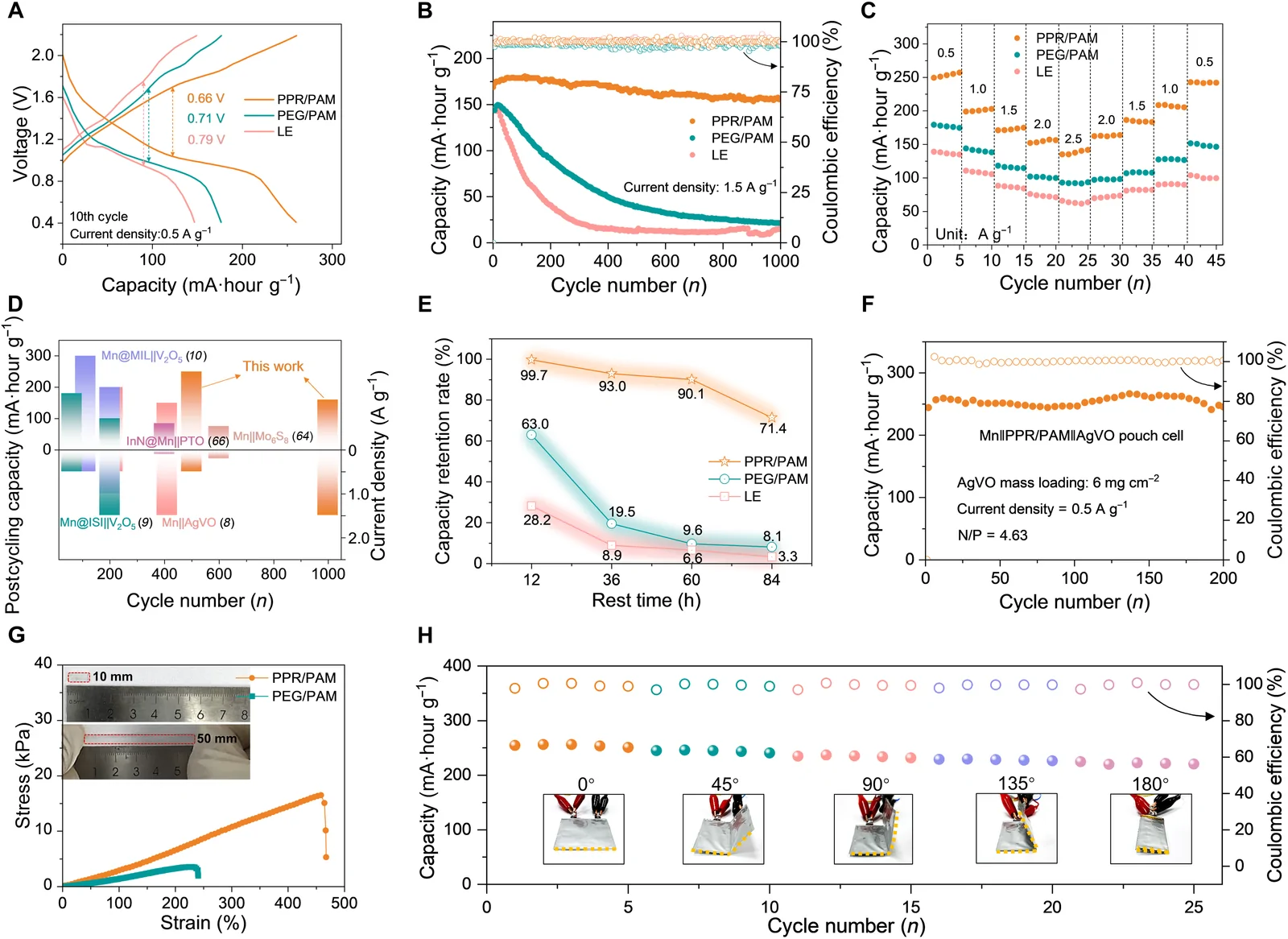
Electrochemical performance of Mn||PPR/PAM||AgVO full cells. (**A**) GCD profiles with various electrolytes. (**B**) Cycling performance at the current density of 1.5 A g^−1^. (**C**) Rate performance. (**D**) Comparison of cyclic performance with previously reported full cells (*8*–*10*, *64*, *66*). (**E**) Capacity retention after 12, 36, 60, and 84 hours of rest. (**F**) Cycling performance of the pouch cell. (**G**) Typical tensile stress-strain curves of PPR/PAM and PEG/PAM, with insets showing digital photos of PPR/PAM under stretching. (**H**) Cycle performance of the pouch cell under continuous mechanical deformation (insets: photos of the pouch cells under different bending angles).

To assess the practical potential of the PPR/PAM electrolyte, quasi-solid-state full cells were assembled using Mn as the anode and Ag_0.11_V_2_O_5_ (AgVO) as the cathode (see fig. S28 for cathode characterizations). From the galvanostatic charge/discharge (GCD) profiles, the Mn||PPR/PAM||AgVO full cell delivers the highest discharge capacity of 260.0 mA·hour g^−1^ at 0.5 A g^−1^, accompanied by the lowest polarization voltage of 0.66 V compared to 0.71 V for PEG/PAM and 0.79 V for LE (Fig. 6A). CV curves of the PPR/PAM full cell exhibit higher current densities than those of the PEG/PAM-based and LE-based cells, indicative of the improved reaction kinetics and Mn redox activity (fig. S29). As expected from previous discussions, the PPR/PAM full cell achieves long-cycle stability and high-rate capability, both much superior to the other two types of full cells with PEG/PAM and LE (Fig. 6, B and C, and fig. S30). Specifically, the capacity retention is above 90% after 500 cycles at 0.5 A g^−1^ and after 1000 cycles at 1.5 A g^−1^. Furthermore, the broad ESW of the PPR/PAM hydrogel enables the operation of a high-voltage Prussian blue analog cathode, Zn_3_(Fe(CN)_6_)_2_, delivering an output voltage of 1.79 V (fig. S31). These performances make our full cells superior to previous reports (Fig. 6D and table S9).

Self-discharge has been one of the major issues in metal aqueous batteries. This is compared between the three systems. Within the range of 12 to 84 hours, the PPR/PAM full cell consistently retains capacity more robustly than the other two. After resting for 84 hours, the charged PPR/PAM full cell maintains a higher open-circuit voltage with 71.4% capacity retention, exceeding 8.1% with LE and 3.3% with PEG/PAM (Fig. 6E and fig. S32).

Moreover, a Mn||PPR/PAM||AgVO pouch cell with an N/P ratio of 4.63 affords a capacity of 251.3 mA·hour g^−1^ at 0.5 A g^−1^ and retains 95.3% of initial capacity after 200 cycles (Fig. 6F and fig. S33). Before assessing flexibility, we measured the mechanical strength of the hydrogels (Fig. 6G). The PPR/PAM hydrogel gives a tensile strength of 16.5 kPa and elongation at break of 458%, ∼4.7 and 2 times those of PEG/PAM (3.5 kPa and 232%, respectively). Under various bending angles of 45°, 90°, 135°, and 180°, the corresponding pouch cells exhibit capacity retention rates of 94.6, 91.0, 88.8, and 86.5%, respectively (Fig. 6H). The device can power an LED (light-emitting diode) lamp at the folded state (fig. S34), manifesting its resilience against severe deformation.

## DISCUSSION

We have demonstrated a PPR/PAM hydrogel electrolyte that can render a water-repulsing solvation environment and ensure highly reversible and uniform Mn plating/stripping. Central to this design is the introduction of 18C6 into polymer chains, which confines Mn ions within their cavities and simultaneously reduces the hydration number from a nominal 5 to 1. Thorough characterizations and calculations lead to the conclusion that this PPR configuration enforces ion migration through a ring-threading hopping process along a compact polymer channel. This PPR/PAM hydrogel electrolyte exhibits a series of contributing metrics, including ionic conductivity of 53.8 mS cm^−1^, Mn^2+^ transference number of 0.75, and tensile strength of 16.5 kPa. Furthermore, the dynamic coordination mechanism in PPR/PAM suppresses the rehydration of Mn ions during migration and hence reduces the solvated H_2_O molecules from decomposition at the Mn surface. As a result, this regulation strategy has enabled efficient Mn deposition while mitigating H_2_O-related side reactions. Electrochemical measurements reveal an average CE of 95% during Mn plating/stripping upon 400 cycles. The Mn||Mn symmetric cell with PPR/PAM presents over 1800 hours life span (0.1 mA cm^−2^ and 0.1 mA·hour cm^−2^), and the Mn||PPR/PAM||AgVO full cells have much elongated cycling life and reduced self-discharge compared to the full cells with the PEG/PAM hydrogel and liquid electrolytes.

This study highlights the importance of hydrogel electrolyte design in stabilizing high-active metal anodes. Although exemplified with Mn in the current study, similar strategies could be explored for metals such as Mg and Al. The limit of this study is the relatively low energy density [low output voltage of 1.0 V for AgVO or low specific capacity of 100 mA·hour g^−1^ for Zn_3_(Fe(CN)_6_)_2_], making the system not more advantageous than the typical Zn metal aqueous batteries. Possible mitigation solutions include using cathodes with higher redox potentials and more active sites. Further designing the polymer framework is also necessary to increase the bound-water content in the hydrogel, along with a widened ESW (*44*).

## MATERIALS AND METHODS

### Materials

The following chemicals were purchased from Aladdin and used as received without further purification: PEG (average number-average molecular weight: 1500 g mol^−1^), 18C6 (AR), AM (AR), ammonium persulfate [(NH_4_)_2_S_2_O_8_; AR], 1-methyl-2-pyrrolidinone (NMP; AR), manganese perchlorate hexahydrate [Mn(ClO_4_)_2_·6H_2_O; reagent grade], vanadium pentoxide (V_2_O_5_; AR), silver nitrate (AgNO_3_; AR), hydrogen peroxide solution (H_2_O_2_; 30 wt % in H_2_O), and polyvinylidene difluoride (PVDF; average weight-average molecular weight: ∼400,000 g mol^−1^). Mn plates (AR; Metallurgical Jingyao Alloy Research Institute of China) were mechanically ground with a grinder (120-grit abrasive wheel) to remove the surface passivation layer and used directly without any cleaning. Ti foil (AR) was obtained from Qingyuan Metal Materials Co. Ltd. Glass microfiber filters (GF/C, 1822-090, Whatman) were used as separators.

### Preparation of PPR/PAM, PAM, PEG/PAM, and LE

PPR/PAM: PEG (1.0 g) and 18C6 (1.0 g) were dissolved in 10 ml of NMP within a glass bottle inside an argon-filled glove box. The mixture was stirred for 48 hours to form a uniform solution, followed by drying in a vacuum oven for another 48 hours to evaporate the solvent, yielding the PPR matrix. Subsequently, 0.8 g of PPR was dispersed in 2.68 ml of deionized water under vigorous stirring. After complete dissolution, 0.8 g of the AM monomer was added and stirred until homogeneous. Then, 1.09 g of Mn(ClO_4_)_2_ was introduced into the solution, followed by the addition of 0.02 g of (NH_4_)_2_S_2_O_8_ as the initiator. The resulting precursor solution was poured into a PTFE mold and polymerized at 70°C for 2 hours to obtain the PPR/PAM hydrogel.

PAM: For comparison, a pure PAM-based hydrogel electrolyte was synthesized via a similar procedure. A 0.8-g AM monomer was dissolved in 2.68 ml of deionized water. Then, 1.09 g of Mn(ClO_4_)_2_ and 0.02 g of (NH_4_)_2_S_2_O_8_ were added. The solution was stirred until homogeneous and polymerized in a PTFE mold at 70°C for 2 hours.

PEG/PAM: 0.8 g of PEG was first dissolved in 2.68 ml of deionized water. Subsequently, 0.8 g of AM, 1.09 g of Mn(ClO_4_)_2_, and 0.02 g of (NH_4_)_2_S_2_O_8_ were added under stirring. The obtained homogeneous solution was transferred to a PTFE mold and polymerized at 70°C for 2 hours.

LE: The liquid electrolyte was prepared by dissolving 1.09 g of Mn(ClO_4_)_2_ into 2.68 ml of deionized water under stirring until a clear solution was formed.

### Preparation of the AgVO cathode

In a typical hydrothermal process, 0.3 g of V_2_O_5_ powder was dispersed in 30 ml of deionized water. Subsequently, 4 ml of H_2_O_2_ was added dropwise to the suspension under magnetic stirring. The mixture was stirred for 30 min at 40°C until the solution turned orange. Then, 28.0 mg of AgNO_3_ was introduced into the solution, and the mixture was stirred at 40°C for a further 30 min. The resulting precursor solution was transferred into a 50-ml Teflon-lined stainless steel autoclave and heated at 200°C for 48 hours. Following a natural cooling period to room temperature, the precipitate was collected by centrifugation, washed repeatedly with ethanol and deionized water, and lastly dried in a vacuum oven at 60°C for 12 hours to obtain the final AgVO product.

### Materials characterization

FTIR spectra were recorded on Bruker VERTEX 70. Solid-state nuclear magnetic resonance (ssNMR) measurements were conducted using a Bruker 600 MHz NMR spectrometer. The ^1^H NMR spectra were recorded under magic angle spinning (MAS) conditions at 8 kHz using a Hahn-echo pulse sequence. Raman spectroscopy was measured by a Via Raman microscope (Renishaw, UK) equipped with a diode-pumped solid-state laser (532 nm, Nd line laser source). TOF-SIMS experiments were performed on ION-TOF GmbH (Münster, Germany) equipped with a 30-keV Bi^3+^ primary ion gun and a 2-keV Cs^+^ sputter gun. The scanning area is 50 μm by 50 μm, and the scanning speed is 1 μm/s. Chemical composition of the samples was obtained by an x-ray photoelectron spectrometer (ESCALAB 250). The morphology of the hydrogel electrolytes and the Mn anodes was detected by a scanning electron microscope (SU 8020, Hitachi, Japan). To investigate the elemental distribution, energy-dispersive x-ray spectroscopy (EDX) was performed on a Tecnai G2 F20 S-TWIN coupled with an EDX analysis system (BRUKER AXS). XRD was used to characterize the by-products on Bruker D8 ADVANCE (Cu Kα, λ = 0.154056 nm). The Mn anodes during cycling were recorded by an optical microscope (Zeiss AXIO Lab A1). The GC measurement was conducted in beaker cells via SHIMADZUGC-2030C. Before the test, the beaker cells were vented with Ar gas for 5 min to remove the air inside. 3D morphology was characterized by a laser microscope (KEYENCE VK-X150). The liquid ion conductivity was tested by a digital conductivity meter (DDBJ-350F, Shanghai Leici Products Marketing Center of China). Typical tensile stress-strain curves of electrolytes were carried out by an electronic universal testing machine (CMT6103, American). To determine the water content, the initial weight (*W*_i_) of the hydrogel electrolyte was measured after preparation, and then it was dried in a vacuum oven at 100°C for 24 hours to reach the final weight (*W*_f_). The final water content was equal to (*W*_i_ − *W*_f_)/*W*_i_ × 100%.

### Isotope calibration experiment

Hydrogel electrolytes were prepared using a 200-μm-thick PTFE mold, with the PEG/PAM and PPR/PAM hydrogel synthesized with either deuterated water (D_2_O) or protiated water (H_2_O). The resulting D_2_O-based and H_2_O-based hydrogel layers were stacked into a symmetric cell. A directional current was then applied for 1, 5, and 50 hours to drive Mn^2+^ migration from the D_2_O layer to the H_2_O layer. Following charging, the H_2_O layer was analyzed by FTIR and Raman spectroscopy to track isotope-specific vibrations.

### Electrochemical characterization

To assemble symmetric cells, two polished Mn plates were used as electrodes. In asymmetric cells, Cu foil is used as the counter electrode. For Mn||AgVO cells, polished Mn was used as the anode, with PPR/PAM, PEG/PAM, or LE as the electrolyte. The cathode was made by casting the slurries including AgVO (70 wt %), Super P (20 wt %), and PVDF (10 wt %) in NMP solution onto Ti foil. The diameter of the cathode was 10 mm, and the areal loading of AgVO was ∼1.0 mg cm^−2^. The symmetric, asymmetric, and normal full cells were assembled in Swagelok molds. For pouch cells, the AgVO cathode was casted on a Ti foil (4 cm by 4 cm) with an areal loading of ∼6 mg cm^−2^. The Mn anode was fabricated by mixing manganese powder (80 wt %), Super P (10 wt %), and PVDF (10 wt %) in NMP solution. The mixture was evenly coated on the Ti foil. Then, the anode, PPR/PAM hydrogel electrolyte, and cathode were combined and packaged.

The GCD tests were carried out on the LAND battery test system. CV, electrochemical impedance spectroscopy (EIS), chronoamperometry (CA) characterization, LSV curves, and linear polarization plots were all recorded by the Biologic VSP electrochemical workstation. As for the LSV test, a three-electrode system was assembled, with bare Mn, Ti foil, and Ag/AgCl as the working electrode, counter electrode, and reference electrode, respectively. For CA and Tafel tests, the Mn metal is used as the working electrode and counter electrode, and Ag/AgCl serves as the reference electrode in a three-electrode system.

The frequency range of the electrochemical impedance test is 10^−1^ to 10^5^ Hz. The corresponding formula for calculating ionic conductivity is

σ=lRA (1)

where l is the thickness of the gel electrolyte, R is the bulk resistance, and A is the contact area of the electrolyte (*45*).

To calculate the Mn^2+^ transference number, *I*-*t* curves of the symmetric cells at a constant potential of 10 mV were measured. The resistances of the cells before and after polarization were measured separately, and the transference number was calculated using Eq. 2

$$t^{+}=\frac{I_{s}\left(∆V-I_{0}R_{0}\right)}{I_{0}\left(∆V-I_{s}R_{s}\right)}$$ (2)

where $I_{0}$ and $I_{s}$ refer the initial and steady-state current, respectively, ∆V is the applied potential, and $R_{0}$ and $R_{s}$ are the initial and steady-state resistances of the cell determined by impedance spectroscopy, respectively (*46*).

The activation energy ($E_{a}$) of Mn^2+^ was calculated by EIS results of symmetric cells at different temperatures, according to the Arrhenius equation

$$\frac{1}{R_{\text{ct}}}=A\text{exp}\left(-\frac{E_{a}}{\mathrm{RT}}\right)$$ (3)

where $R_{\text{ct}}$ is the interfacial resistance, A is the finger front factor, and $E_{a}$, R, and T are the activation energy, molar gas constant, and absolute temperature, respectively (*47*).

### MD simulations

We performed MD simulations of bulk electrolytes using the GROMACS2024 package (*48*). Initial configurations were generated with Packmol (*49*). The organic species and nonmetal ions were modeled using the GAFF2 force field (*50*) with parameters generated via the Sobtop and Multiwfn software (*51*), whereas metal ions were described using the Merz-Kollman (*52*) Optimized Potentials for Liquids (OPC3) force field (*53*). Atomic charges were calculated in the ORCA (*54*, *55*) software at the B3LYP level of theory with the def2TZVP basis set and then fitted to obtain RESP (*56*) atomic charges by matching the ESP using the Multiwfn software. The model with the scaled charges by 0.75 for charged ions was used to account for electronic screening. Nonbonded interactions were treated with a Lennard-Jones potential (cutoff = 1.2 nm) for van der Waals forces and the particle mesh Ewald (PME) method for long-range electrostatics (rcoulomb = 1.2 nm). All systems were first energy minimized using the steepest descent algorithm to alleviate unfavorable contacts. Preequilibration was conducted in the NPT ensemble at 298.15 K and 1.01325 bar using the V-rescale thermostat and Berendsen barostat for 5 ns (1 fs per step). Following equilibration, production runs were performed in the NVT ensemble using a leapfrog integration scheme with a time step of 1 fs over 10 × 10^6^ steps (10 ns) at the same pressure and at 298.15 K. The equilibrium of the system is validated using the radius of gyration (*R*_g_) of the polymer components (PAM, PEG, and the 18C6 ligand) and the mean square displacement (MSD) of water molecules (fig. S35). Periodic boundary conditions were applied in all Cartesian directions, and the output (log, energy, and compressed coordinates) was recorded at regular intervals to ensure robust statistical sampling.

### DFT calculations

All quantum chemical calculations were performed using the Gaussian 16 suite of programs (*57*). The geometries of the manganese-aqua complex in both the doublet (spin multiplicity = 2) and sextet (spin multiplicity = 6) spin states were fully optimized without symmetry constraints. Geometry optimizations were carried out at the DFT level using the hybrid functional B3LYP (*58*), including the D3BJ empirical dispersion correction. The 6-31+G(d) all-electron basis set was used for C, N, O, and H atoms, whereas the LANL2DZ basis set with its corresponding effective core potential (ECP) was used for the manganese atom to account for scalar relativistic effects (*59*, *60*).

Following geometry optimization, vibrational frequency calculations were performed at the same level of theory to confirm that the optimized structures correspond to true local minima on the potential energy surface, as evidenced by the absence of imaginary frequencies. Wave function stability analysis was also conducted to ensure the reliability of the self-consistent field (SCF) solutions for both spin states.

To evaluate the binding affinity between water molecules and the Mn^2+^ ion, subsequent single-point energy calculations were carried out at the same B3LYP-D3BJ/6-31+G(d)&LANL2DZ level of theory as the geometry optimization to obtain accurate total energies. The binding energy between the adsorbate and the substrate can be calculated using the following equation

$$∆E_{\text{bind}}=E_{\text{adsorbate}@\text{substrate}}-E_{\text{substrate}}-E_{\text{adsorbate}}$$ (4)

In Eq. 4, $E_{\text{adsorbate}@\text{substrate}}$ and $E_{\text{substrate}}$ represent the total energies of the substrate with and without the adsorption of adsorbate, respectively. $E_{\text{adsorbate}}$ is the total energy of the adsorbate. According to this equation, a negative adsorption energy corresponds to a stable adsorption structure.

### Computational details of Mn^2+^ migration energy

Initial geometries of the reactant and product were preoptimized using the semiempirical extended tight-binding method GFN2-xTB (*61*), which provides efficient and reliable starting structures for reaction pathway calculations. The minimum energy pathway was then explored with the climbing-image nudged elastic band (CI-NEB) method as implemented in ORCA 6.1.0 (*62*). Eight intermediate images were generated by interpolation between the optimized endpoints. A standard NEB preoptimization was first carried out, followed by CI-NEB, in which one image climbs toward the transition state. The calculations used energy-weighted band optimization, DNEB perpendicular spring correction, and SCF convergence checks at each step. Convergence criteria included a maximum force threshold of 6.0 × 10^−4^ Ha/bohr, a maximum displacement of 0.12 Å per step, and up to 500 optimization cycles. The resulting minimum energy pathway and relative energy profile were analyzed, and the structures/trajectories were visualized with VMD 1.9.3 (Visual Molecular Dynamics) (*63*).
