# Supplementary material for: Long-duration aqueous manganese metal anode by hydrogel solvation regulation
Source: Sci Adv. 2026 Sep 25;12(39):eaeg1770. doi: 10.1126/sciadv.aeg1770 (PMC13614402; doi:10.1126/sciadv.aeg1770)
Supplement: Supplementary file 1 [file sciadv.aeg1770_sm.pdf]

Science Advances
AAAS

Supplementary Materials for

Long-duration aqueous manganese metal anode by hydrogel solvation regulation

Hongbao Zheng *et al.*

Corresponding author: Dong Zhang, dongzhang@jlu.edu.cn; Heng Jiang, jiangheng@jlu.edu.cn; Fei Du, dufei@jlu.edu.cn

Sci. Adv. **12**, eaeg1770 (2026)
DOI: 10.1126/sciadv.aeg1770

This PDF file includes:

Figs. S1 to S35
Tables S1 to S9

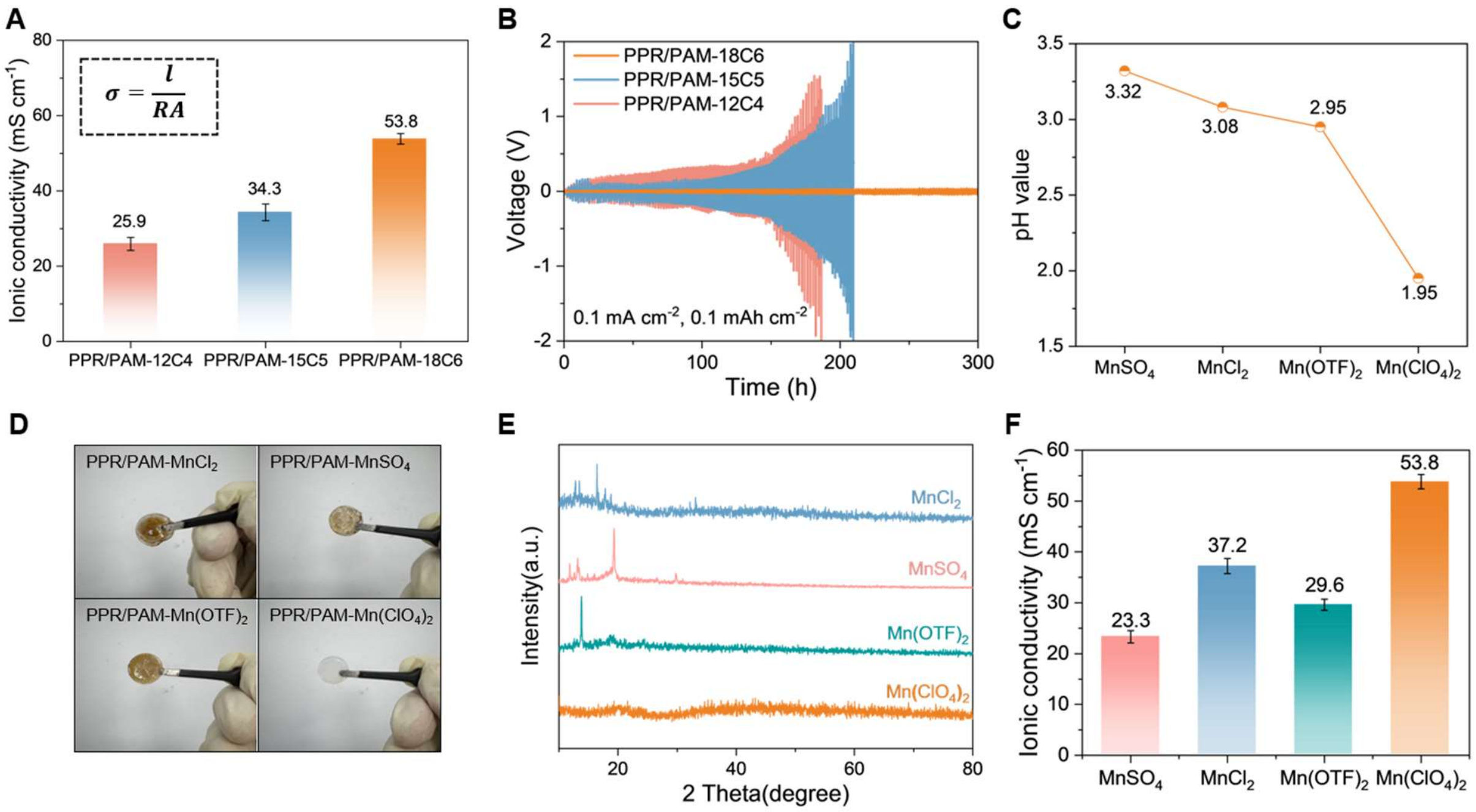

Fig. S1. Optimization of PPR/PAM hydrogel. (**A**) The ionic conductivities of PPR/PAM hydrogel with various crown ethers. (**B**) Cyclic stability of Mn||Mn symmetric cells based on PPR/PAM hydrogel with various crown ethers at 0.1 mA cm^{-2} and 0.1 mAh cm^{-2}. (**C**) pH value of different manganese salts. (**D**) Digital photographs of PPR/PAM hydrogel electrolytes with various manganese salts. (**E**) XRD patterns of different manganese salts. (**F**) The ionic conductivities of PPR/PAM hydrogel with various manganese salts.

Note: First, we systematically compared three crown ethers, i.e., 12C4, 15C5, and 18C6, under identical conditions. Among them, the PPR/PAM-18C6 hydrogel exhibited the highest average ionic conductivity (53.8 mS cm^{-1}) (**Fig. S1A)** and enabled the most stable cycling of Mn||Mn symmetric cells over 300 h with the smallest overpotential (**Fig. S1B**). Consequently, 18C6 was selected as the optimal macrocyclic component. Second, the choice of perchlorate salt was dictated by the polymerization process. Among $MnCl_2$, $MnSO_4$, $Mn(OTf)_2$, and $Mn(ClO_4)_2$, only perchlorate generated a sufficiently acidic environment (**Fig. S1C**). According to the Mn–H_2O Pourbaix diagram, this acidity effectively suppresses the oxidation of Mn^{2+} to higher valence states under ambient atmosphere at 70 °C. The other salts led to brownish discoloration and produced manganese oxide impurities, as confirmed by powder X-ray diffraction (**Fig. S1D** and **E**). The perchlorate-based route yielded a pure pseudorotaxane framework without detectable oxide phases and the as-prepared hydrogel delivered the highest ionic conductivity (53.8 mS cm^{-1}, **Fig. S1F**).

Thus, $Mn(ClO_4)_2$ was essential for maintaining Mn^{2+} stability and framework purity. Based on this comprehensive investigation, 18C6 and $Mn(ClO_4)_2$ were selected for optimal electrochemical performance.

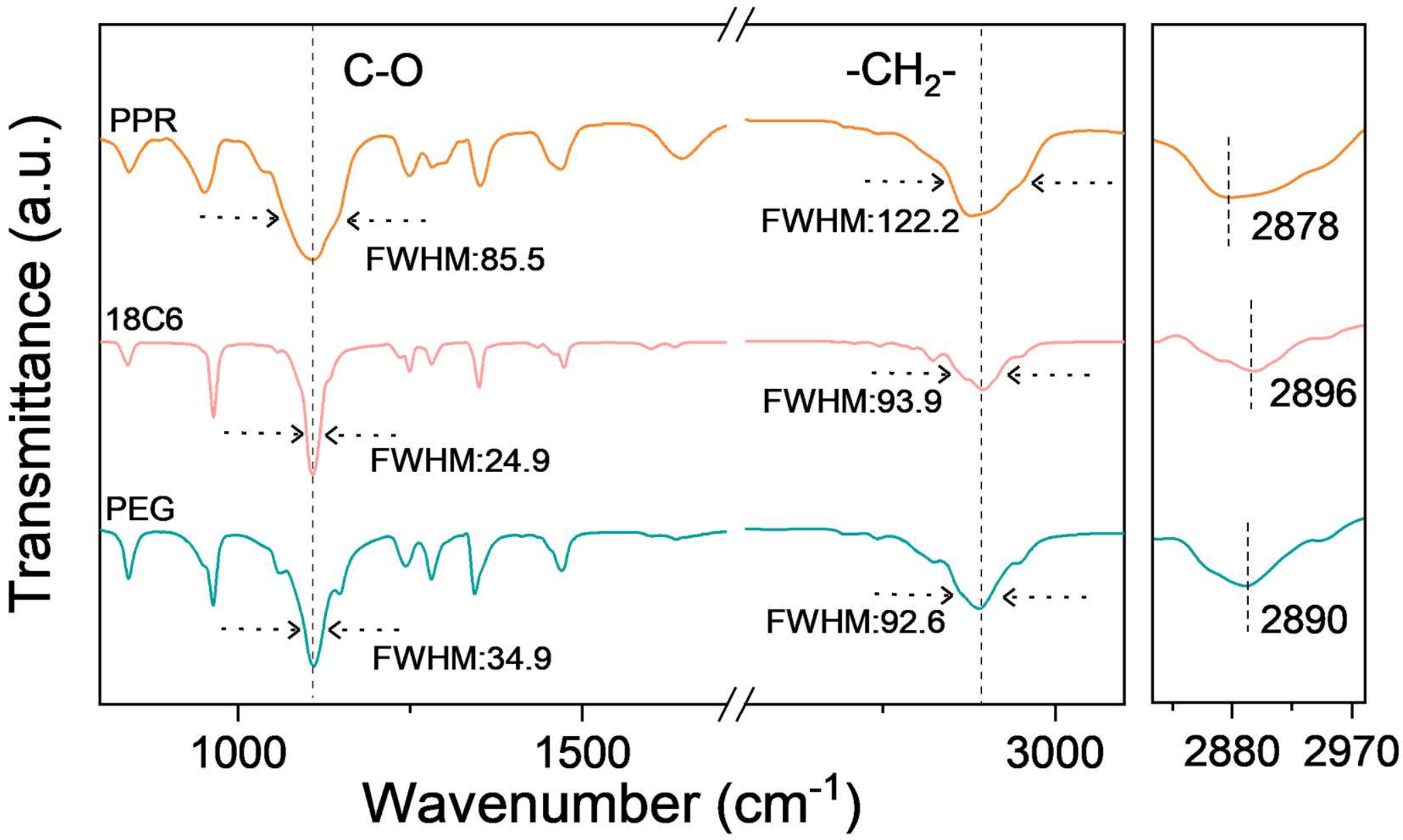

Fig. S2. FTIR spectra of PEG, 18C6, and PPR. The right figure shows a magnified region of the -CH_2- bonds in the samples.

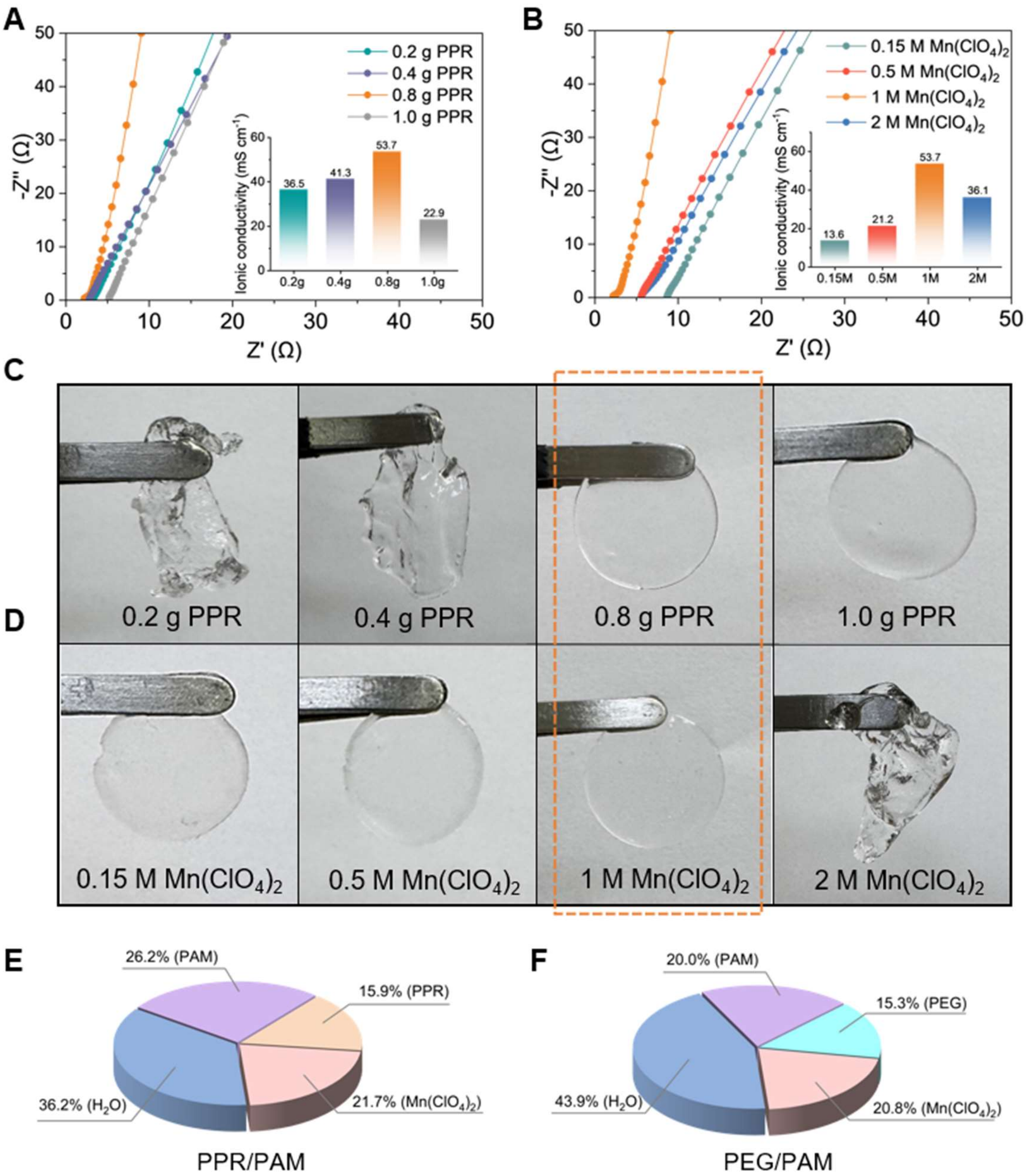

Fig. S3. Optimization of hydrogel electrolyte composition. (**A**) Ionic conductivities of hydrogel electrolytes with various PPR contents. (**B**) Ionic conductivities of hydrogel electrolytes with various Mn salt contents. (**C**) Digital photographs of hydrogel electrolytes with various PPR contents. (**D**) Digital photographs of hydrogel electrolytes with various Mn salt contents. The composition of (**E**) PPR/PAM and (**F**) PEG/PAM hydrogels.

Note: The composition of the PPR/PAM hydrogel was optimized by adjusting the PPR content and Mn salt concentration to achieve both high ionic conductivity and good mechanical stability. PPR provides fast transport pathways for Mn^{2+}, whereas the Mn salt concentration governs the

hydrogel's crosslinking integrity and the availability of charge carriers. We first varied the PPR content while holding the Mn salt at ~1 M (1.09 g). As the PPR content increased to 0.8 g, the ionic conductivity rose steadily to a maximum of 53.7 mS cm^{-1} (**Fig. S3A**), and the hydrogel network became more stable (**Fig. S3C**). This improvement arises from the robust, interconnected network formed by PPR, further facilitating Mn^{2+} migration. However, increasing the PPR content beyond 0.8 g caused the conductivity to decline, due to an excess of coordination sites that immobilize Mn^{2+}, reduce relative water content, and restrict chain mobility. We then fixed the PPR content at 0.8 g and varied the Mn salt concentration. Both insufficient and excessive Mn salt concentrations lead to low conductivity (**Fig. S3B**). A low salt concentration limits charge carriers and ion-hopping sites, while an excess salt concentration promotes ion aggregation and gradually softens the PPR/PAM hydrogel due to Mn^{2+} competing with PAM and PPR for crosslinking sites, eventually leading to network collapse (**Fig. S3D**). Therefore, achieving rapid Mn^{2+} conduction requires a delicate balance between carrier concentration and network stability. This optimum is achieved with 0.8 g of PPR and 1.09 g of Mn salt, providing both high ionic conductivity and excellent physical integrity. The optimized compositions of the PPR/PAM and PEG/PAM hydrogels are summarized in **Fig. S3E** and **F**.

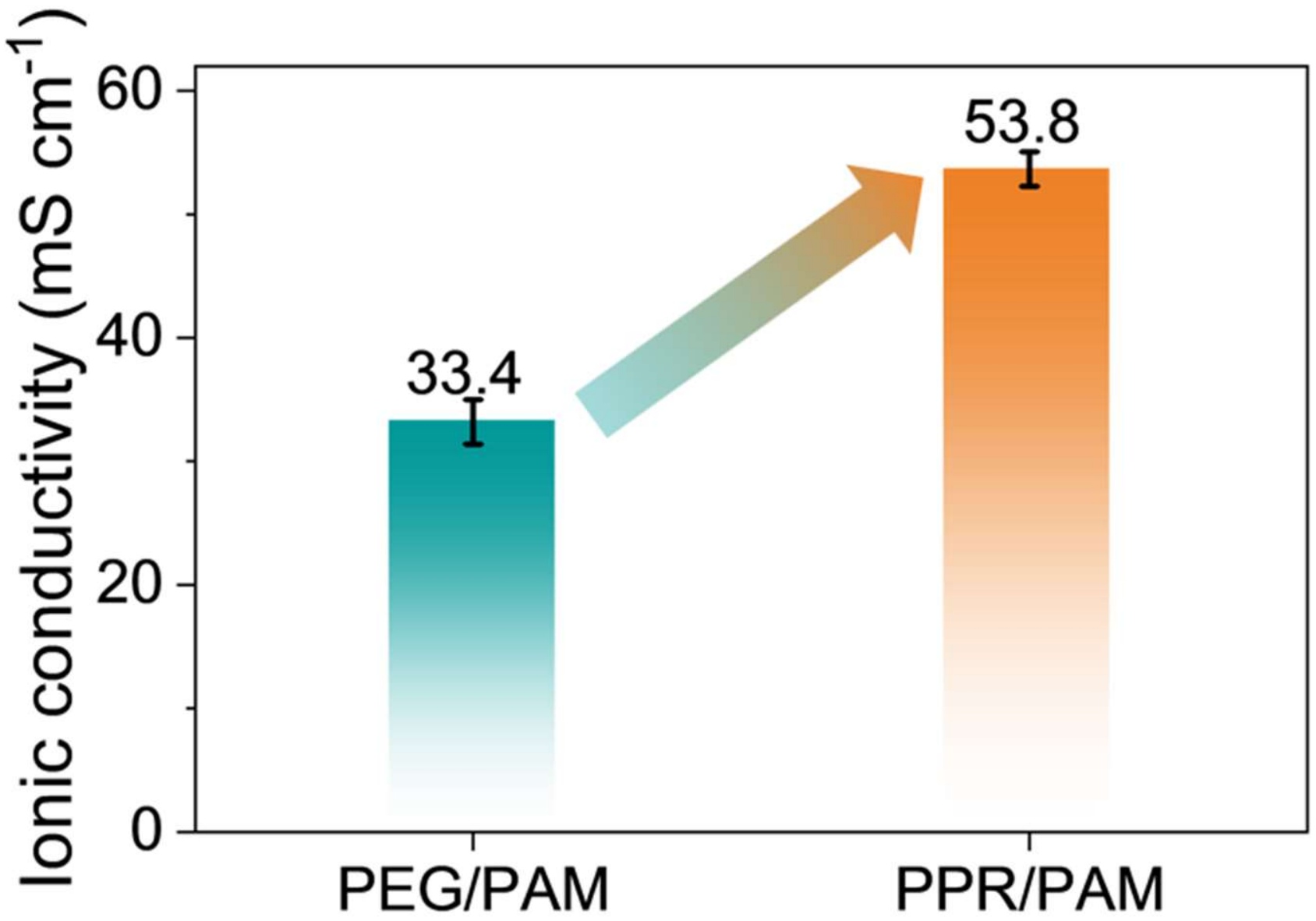

Fig. S4. The ionic conductivities of PPR/PAM and PEG/PAM.

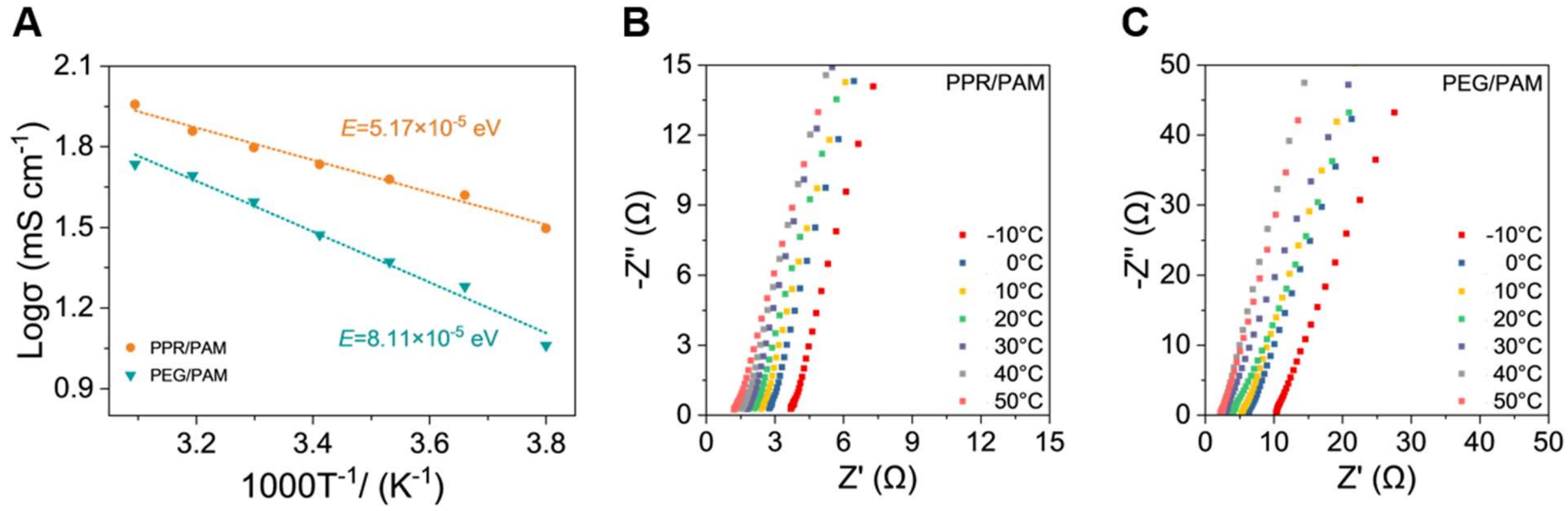

Fig. S5. Temperature-dependent electrochemical properties. (**A**) Ionic conductivity-temperature curves of PPR/PAM and PEG/PAM. EIS of the SS||SS symmetric battery with (**B**) PPR/PAM and (**C**) PEG/PAM at different temperatures.

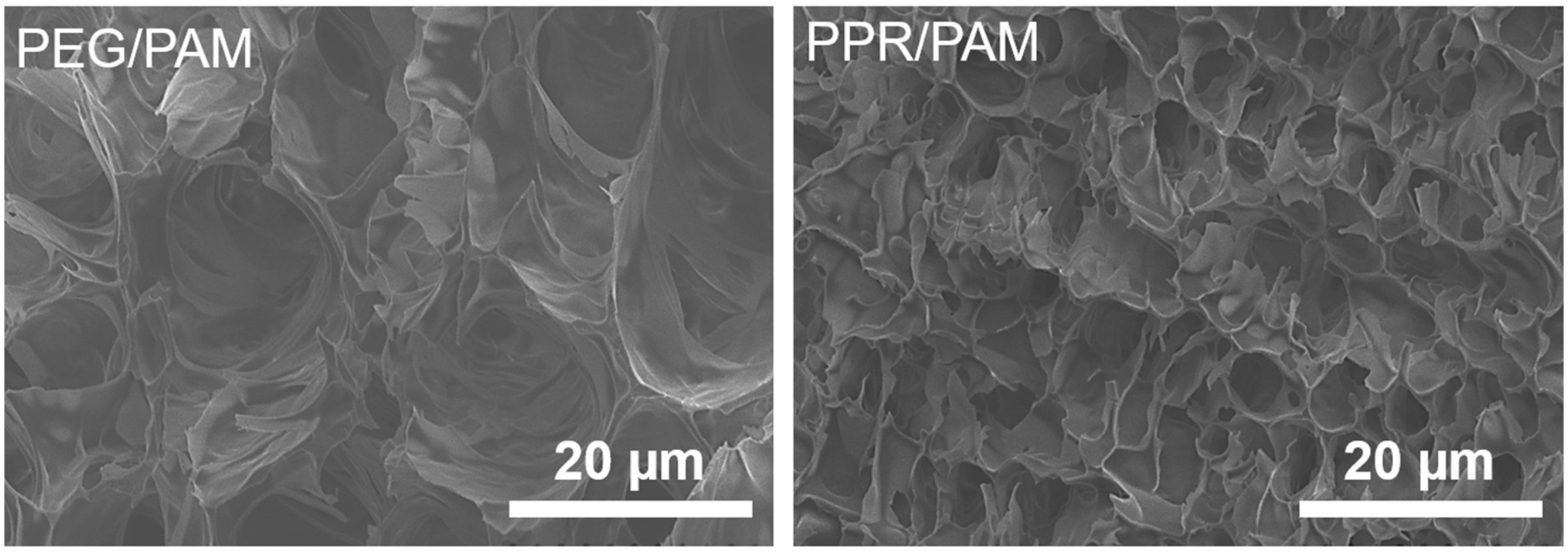

Fig. S6. The cross-sectional SEM images of freeze-dried PEG/PAM and PPR/PAM (different magnification from the surface images shown in Fig. 2D).

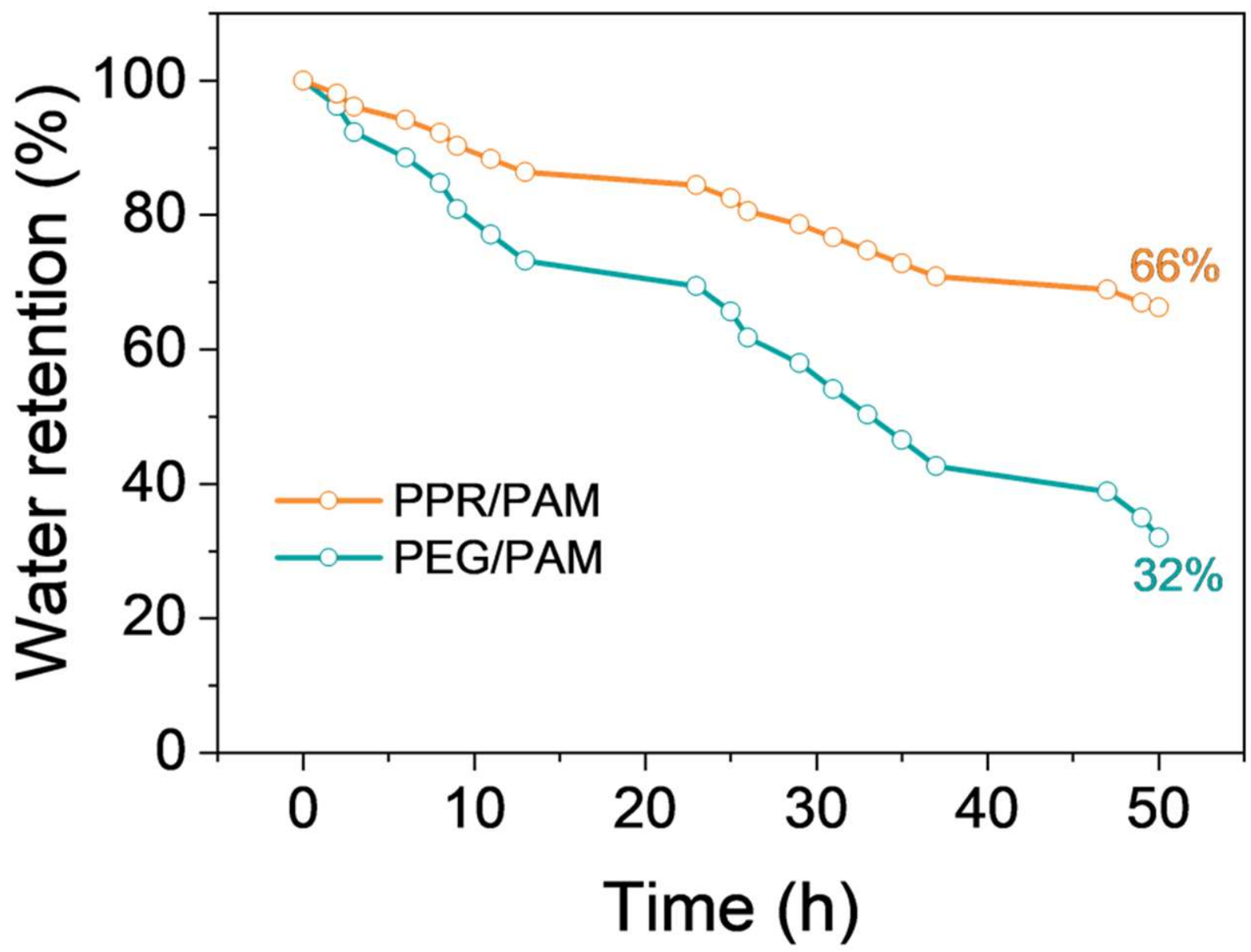

Fig. S7. Water-retention tests of PPR/PAM and PEG/PAM.

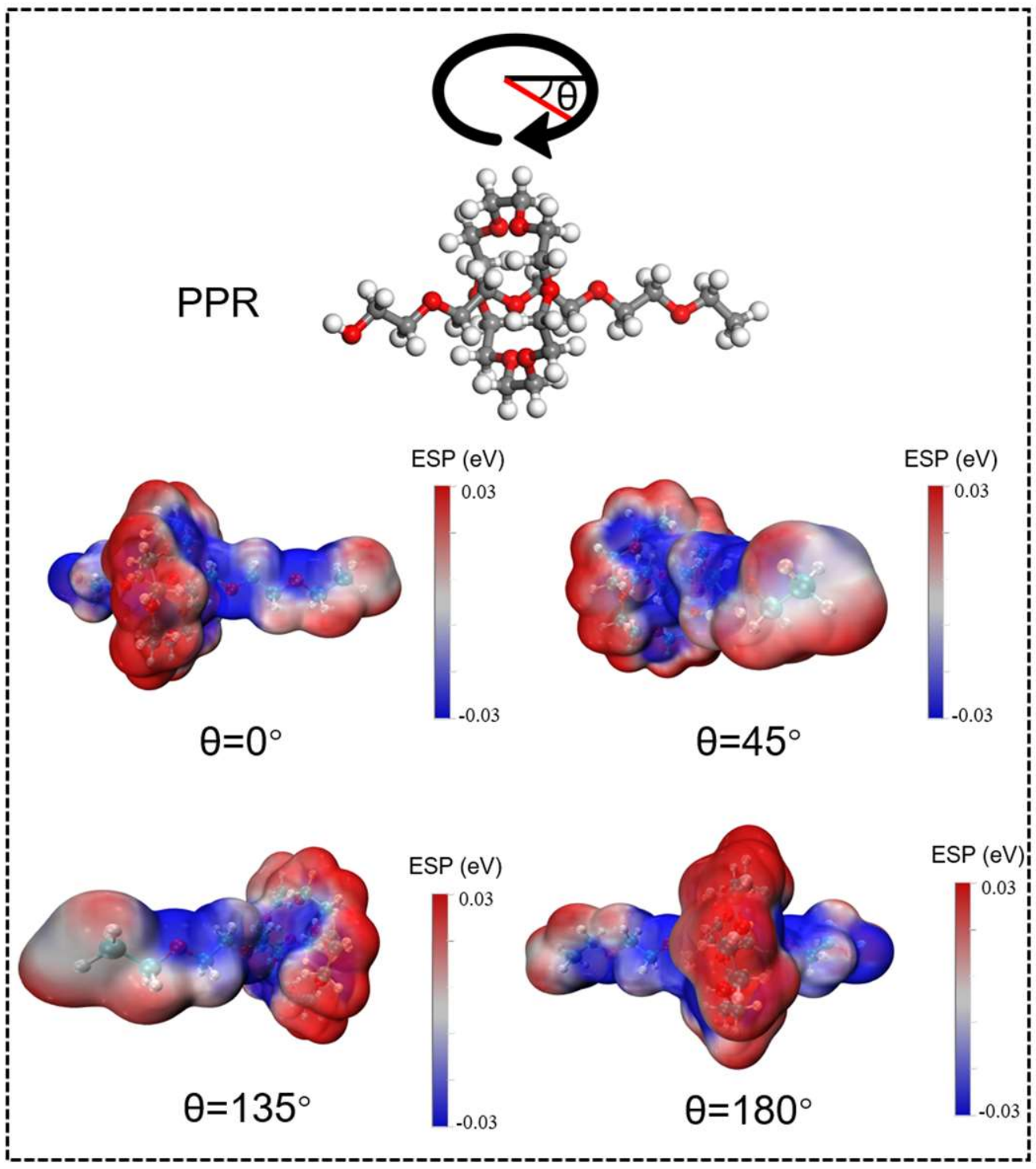

Fig. S8. ESP mapping of PPR structure rotating at different angles.

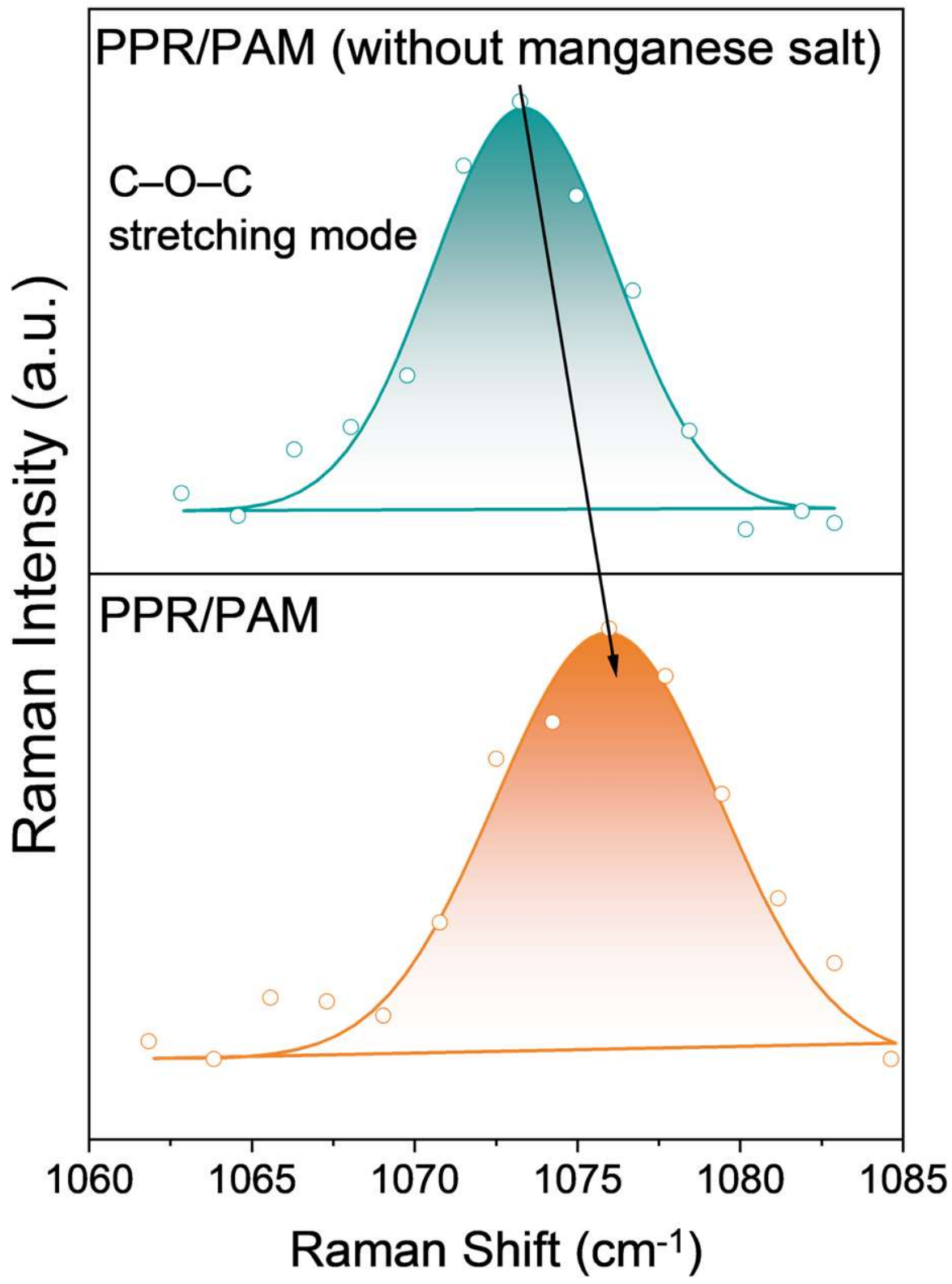

Fig. S9. Raman spectra of PPR/PAM before and after adding Mn salt.

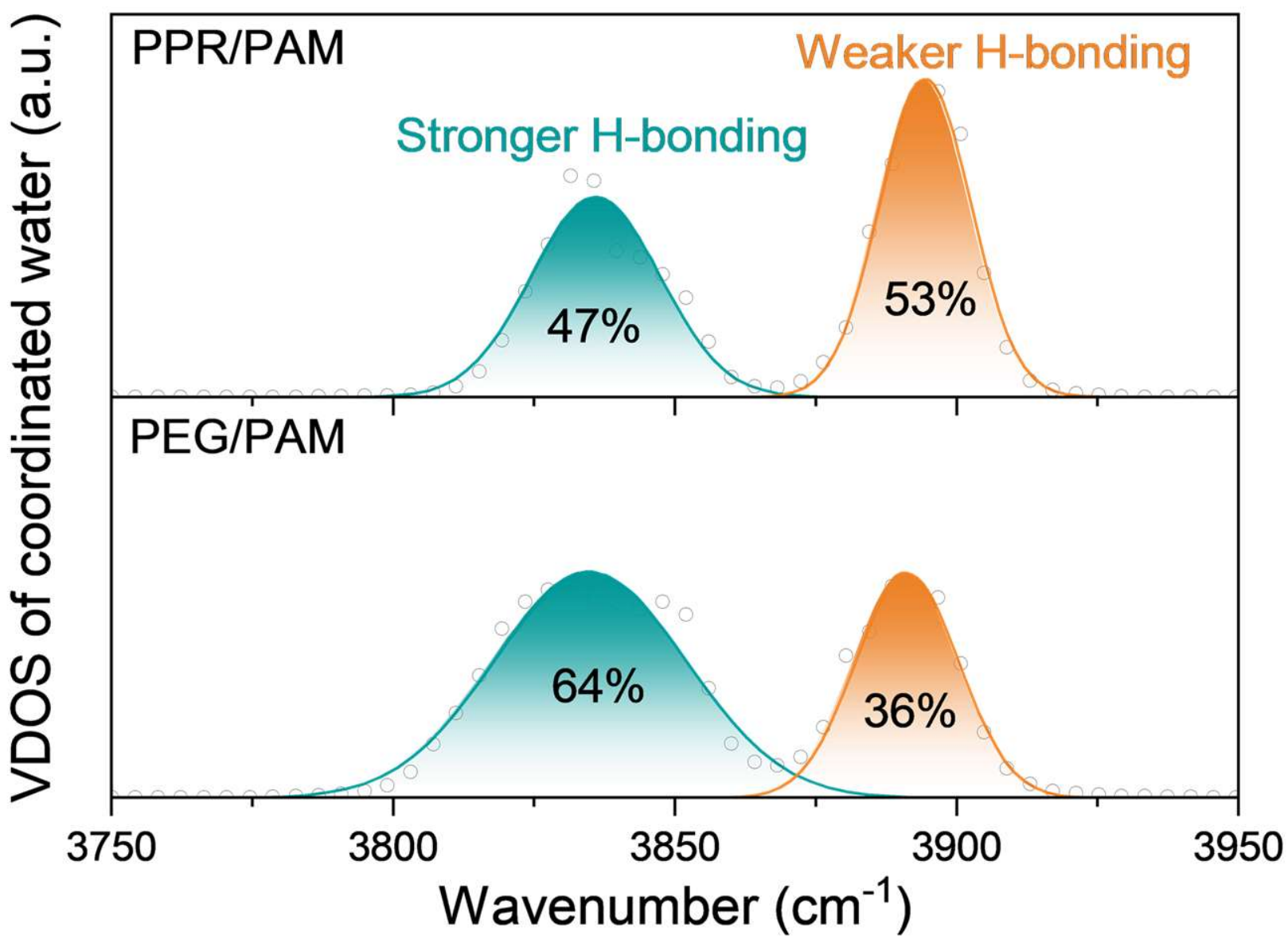

Fig. S10. VDOS of coordinated water in PPR/PAM and PEG/PAM.

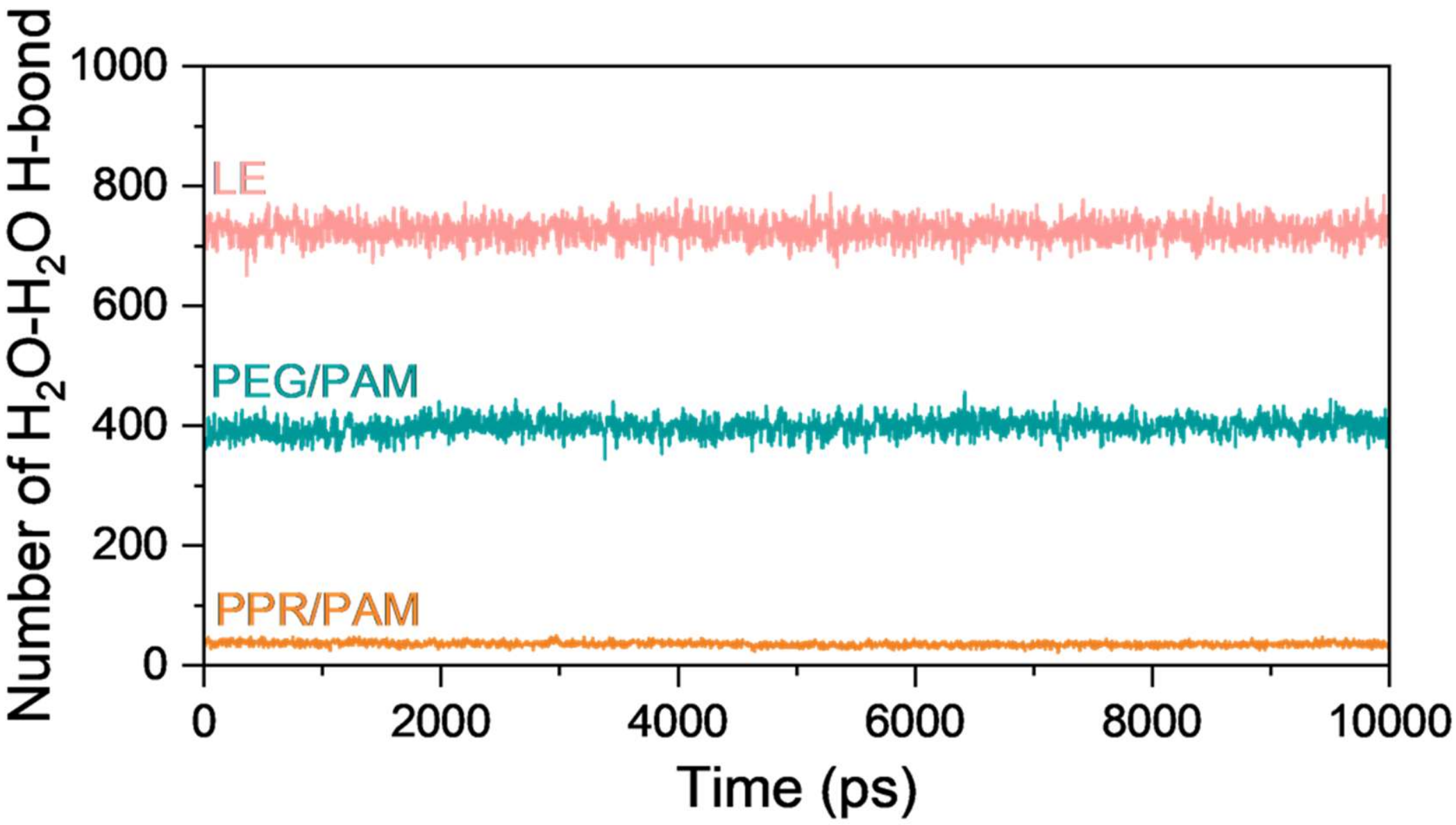

Fig. S11. The number of H_2O-H_2O hydrogen bonds in the three-electrolyte system achieved by MD simulation.

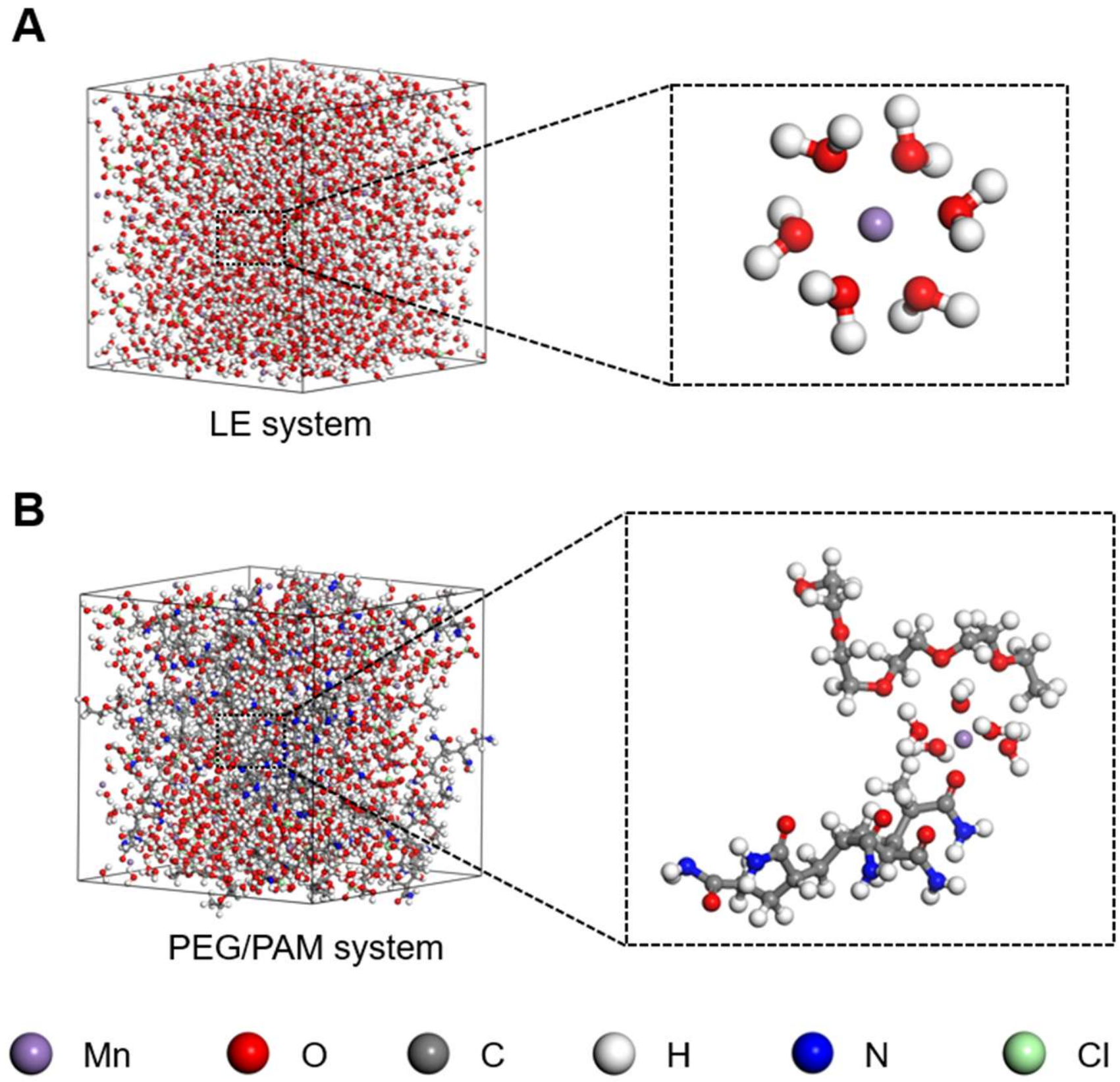

Fig. S12. Solvation structure from MD simulation. 3D snapshot and the enlarged image of the representative solvation structure for (**A**) LE and (**B**) PEG/PAM.

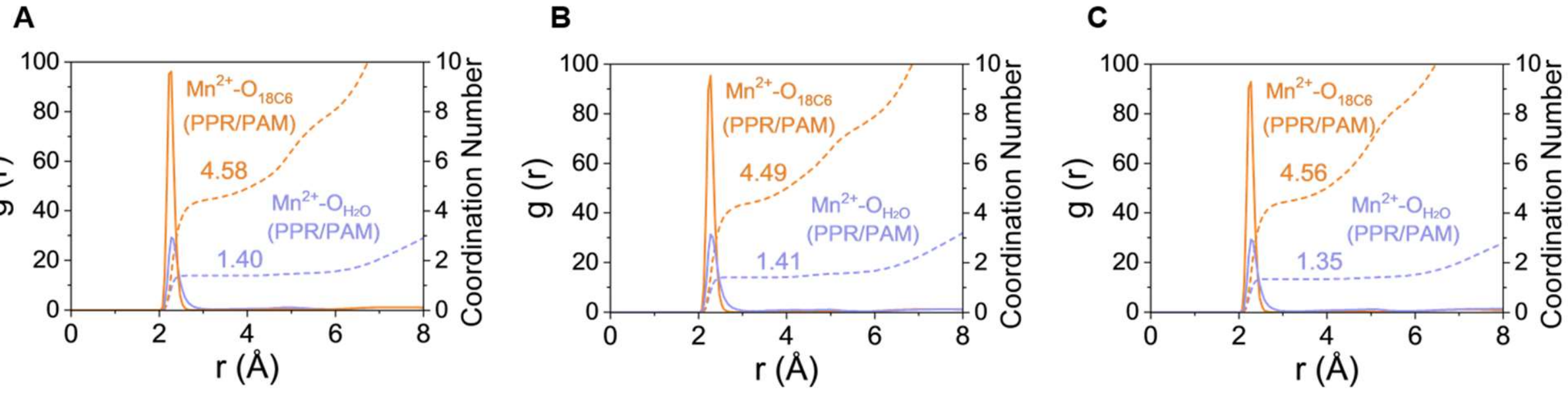

Fig. S13. RDFs and integral curves of Mn^{2+}-O_{H2O} and Mn^{2+}-O_{18C6} in PPR/PAM.

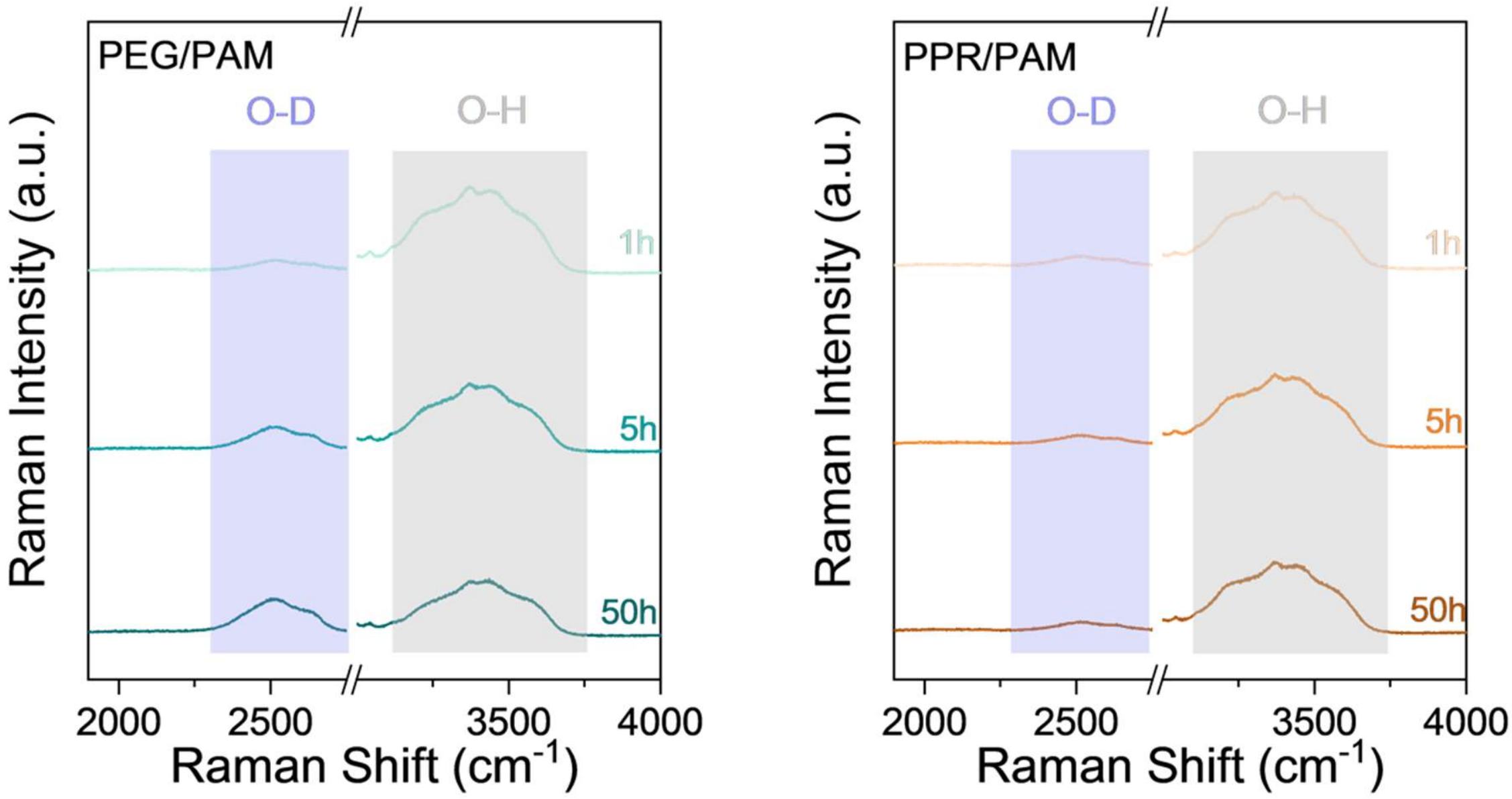

Fig. S14. Raman spectral fitting of O-H and D-H vibrations in different electrolytes.

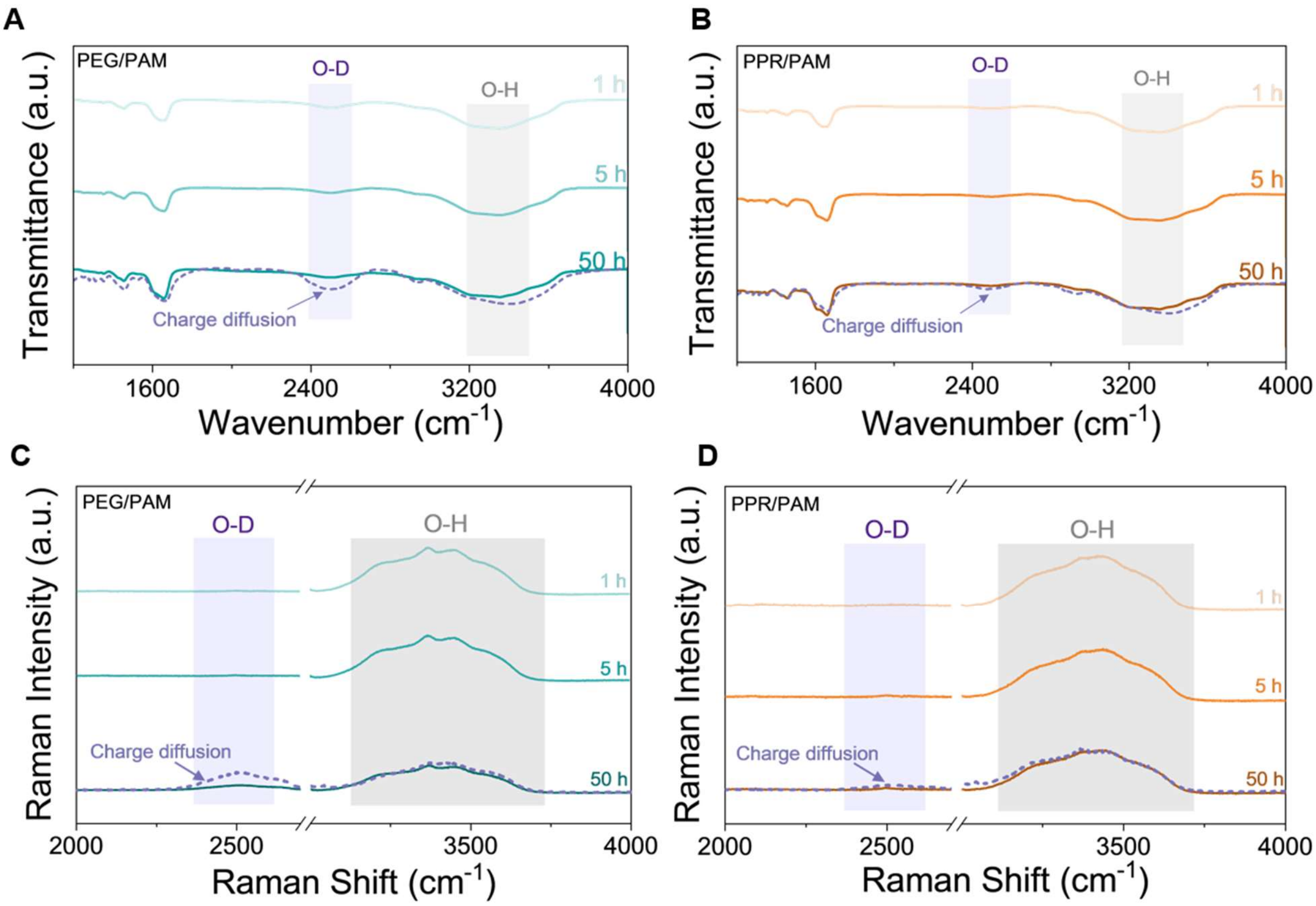

Fig. S15. Static experiment of isotope labeling. FTIR spectra of the upper layer of the H_2O-based electrolyte in (**A**) PEG/PAM and (**B**) PPR/PAM systems. Raman spectra of the upper layer of the H_2O-based electrolyte in (**C**) PEG/PAM and (**D**) PPR/PAM systems.

Note: To investigate the passive water diffusion, we measured FTIR and Raman spectra of both H_2O-hydrogels in contact with the D_2O-hydrogel under static conditions (zero applied potential) for up to 50 h, using the same exposure geometry as in the original electromigration experiment. As shown in **Fig. S15**, no detectable O-D stretching band (~2500 cm^{-1}) appears throughout the entire 50 h period for the PPR/PAM system. This confirms that passive Fickian diffusion of D_2O into PPR/PAM is negligible on the timescale of resting experiments. In contrast, a weak but detectable O-D stretching band emerges after resting for 50 h, indicating measurable D_2O diffusion in the PEF/PAM system. However, this signal is substantially weaker than that of the isotope-labeling experiment under an electric field. This reduction directly demonstrates that the applied field drives additional D_2O transport beyond passive diffusion, which we attribute to Mn^{2+} migrating through the matrix while dragging its coordinated water molecules.

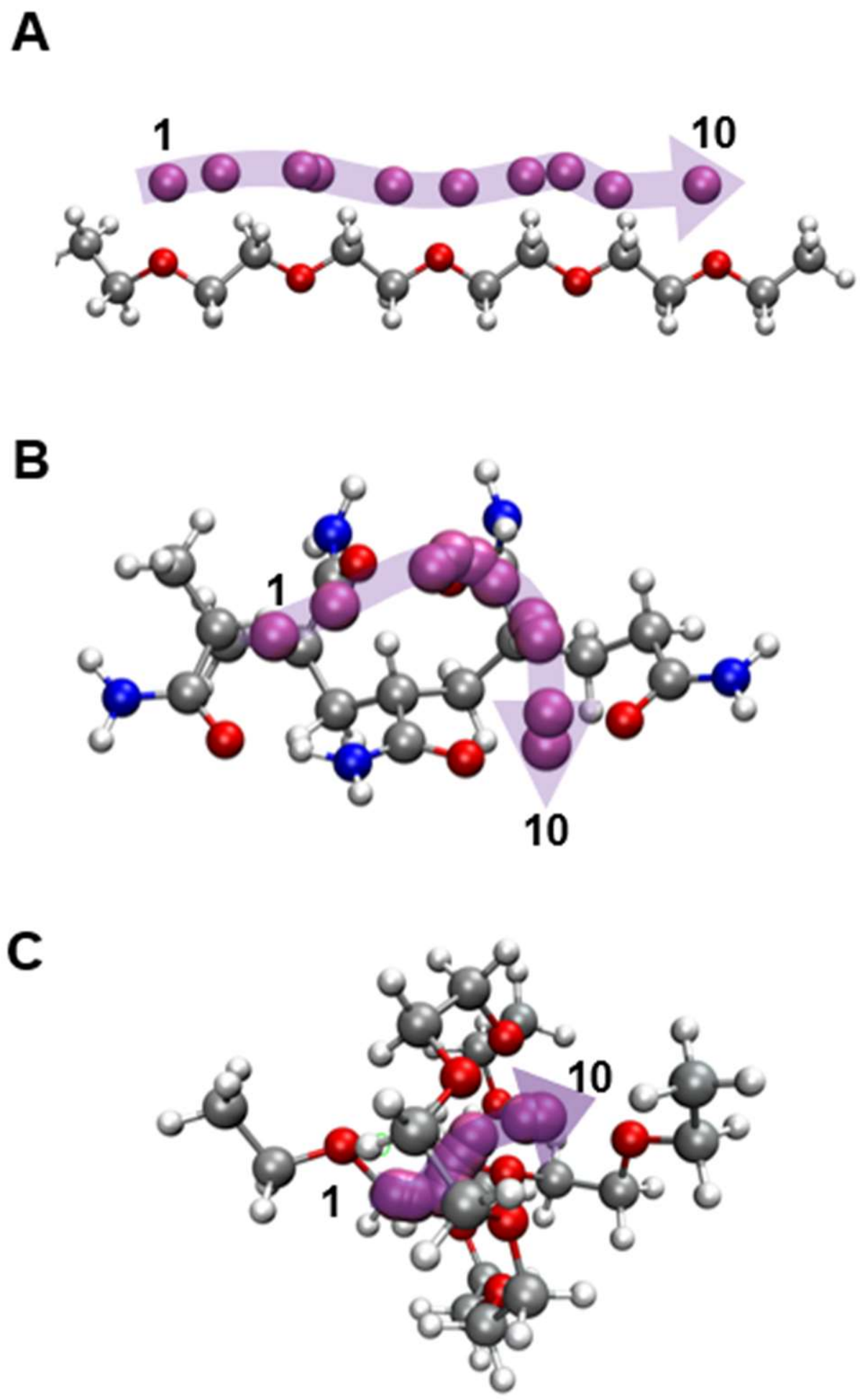

Fig. S16. Migration pathway of Mn^{2+} along different polymer chains from CI-NEB calculation. (**A**) PEG. (**B**) PAM. (**C**) PPR.

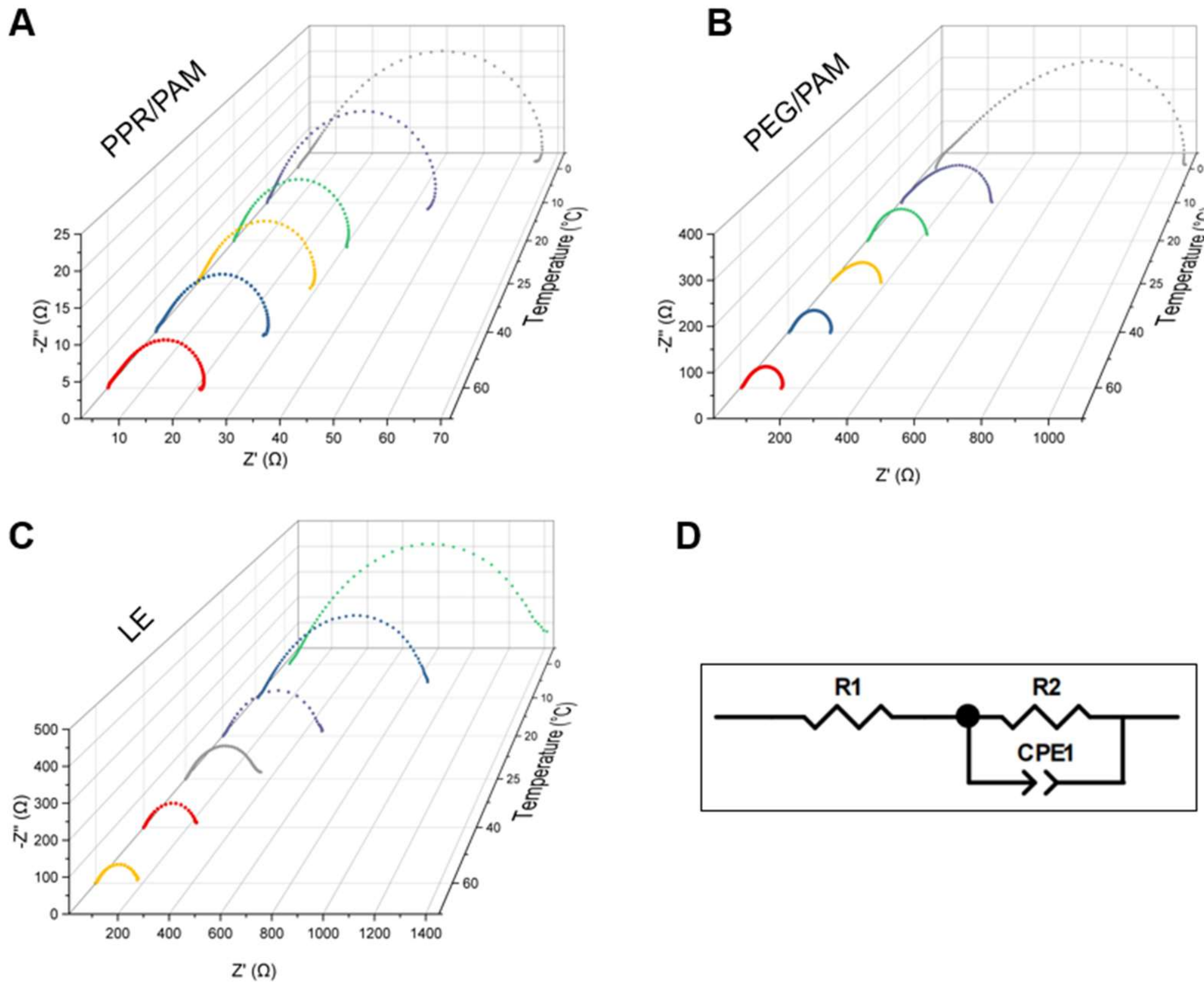

Fig. S17. EIS analysis of symmetric cells. (**A**) Mn||PPR/PAM||Mn at different temperatures. (**B**) Mn||PEG/PAM||Mn at different temperatures. (**C**) Mn||LE||Mn at different temperatures. (**D**) The equivalent circuit diagram of EIS spectra.

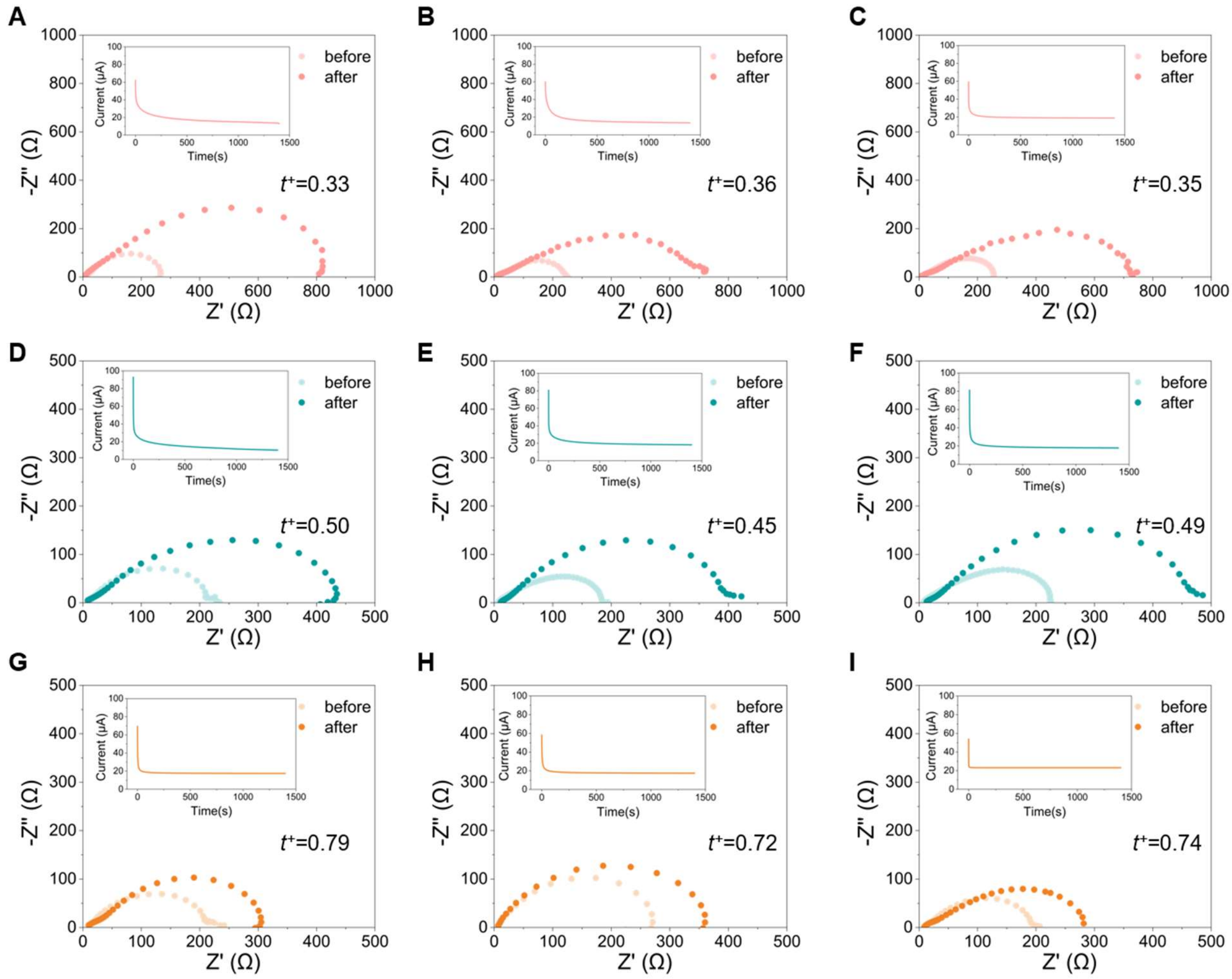

Fig. S18. EIS spectra and current-time plots before and after polarization. (**A-C**) LE electrolyte. (**D-F**) PEG/PAM hydrogel electrolyte. (**G-I**) PPR/PAM hydrogel electrolyte. (Each measurement was repeated three times, and the averaged results were presented in the main text.)

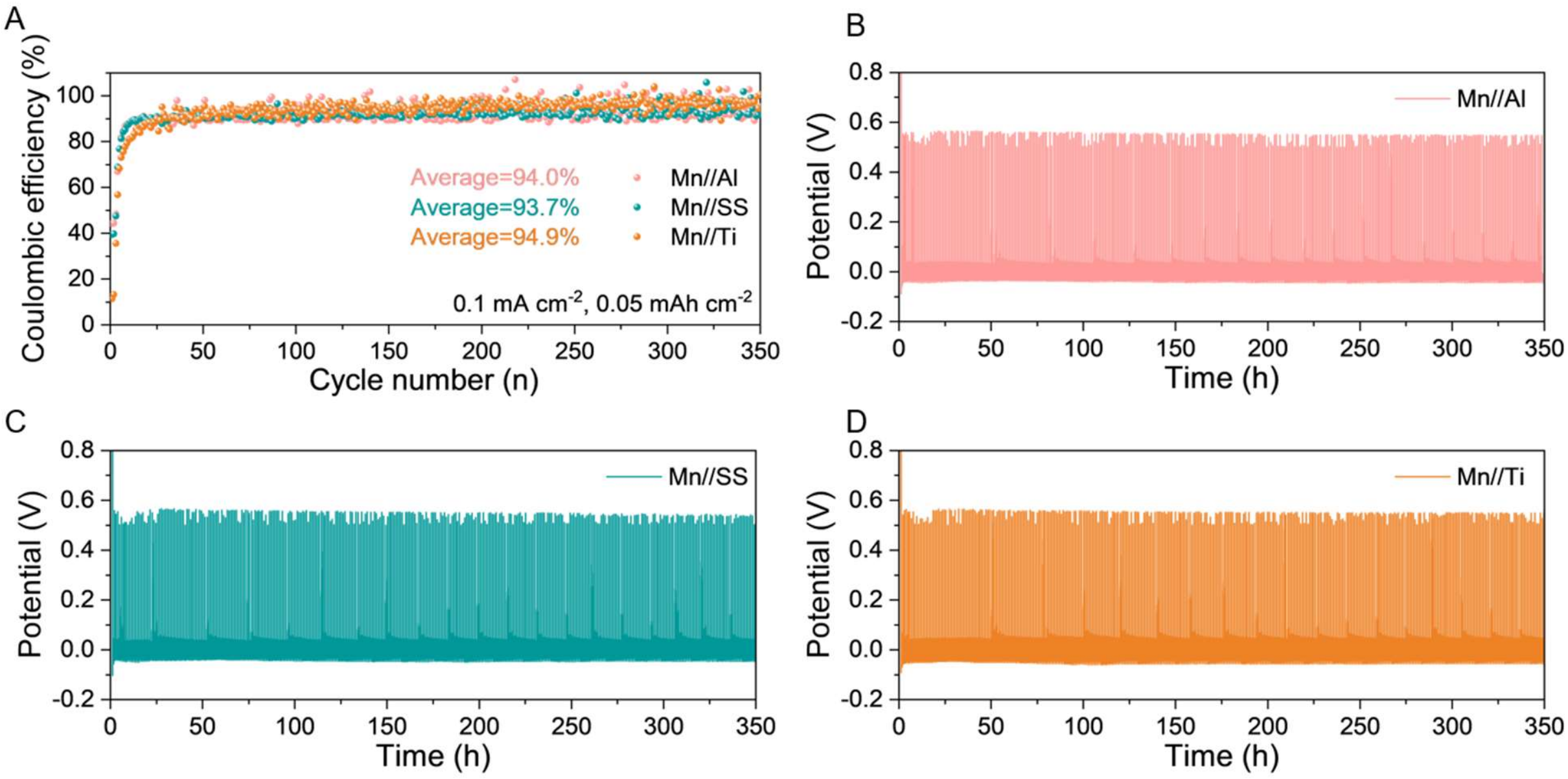

Fig. S19. Mn electroplating/stripping test of different current collectors containing PPR/PAM hydrogel electrolyte. (**A**) Coulombic efficiency and (**B-D**) Potential profiles for asymmetric cells with different collectors, including Al, SS, and Ti, cycled in the PPR/PAM hydrogel electrolyte under 0.1 mA cm^{-2} and 0.05 mAh cm^{-2}.

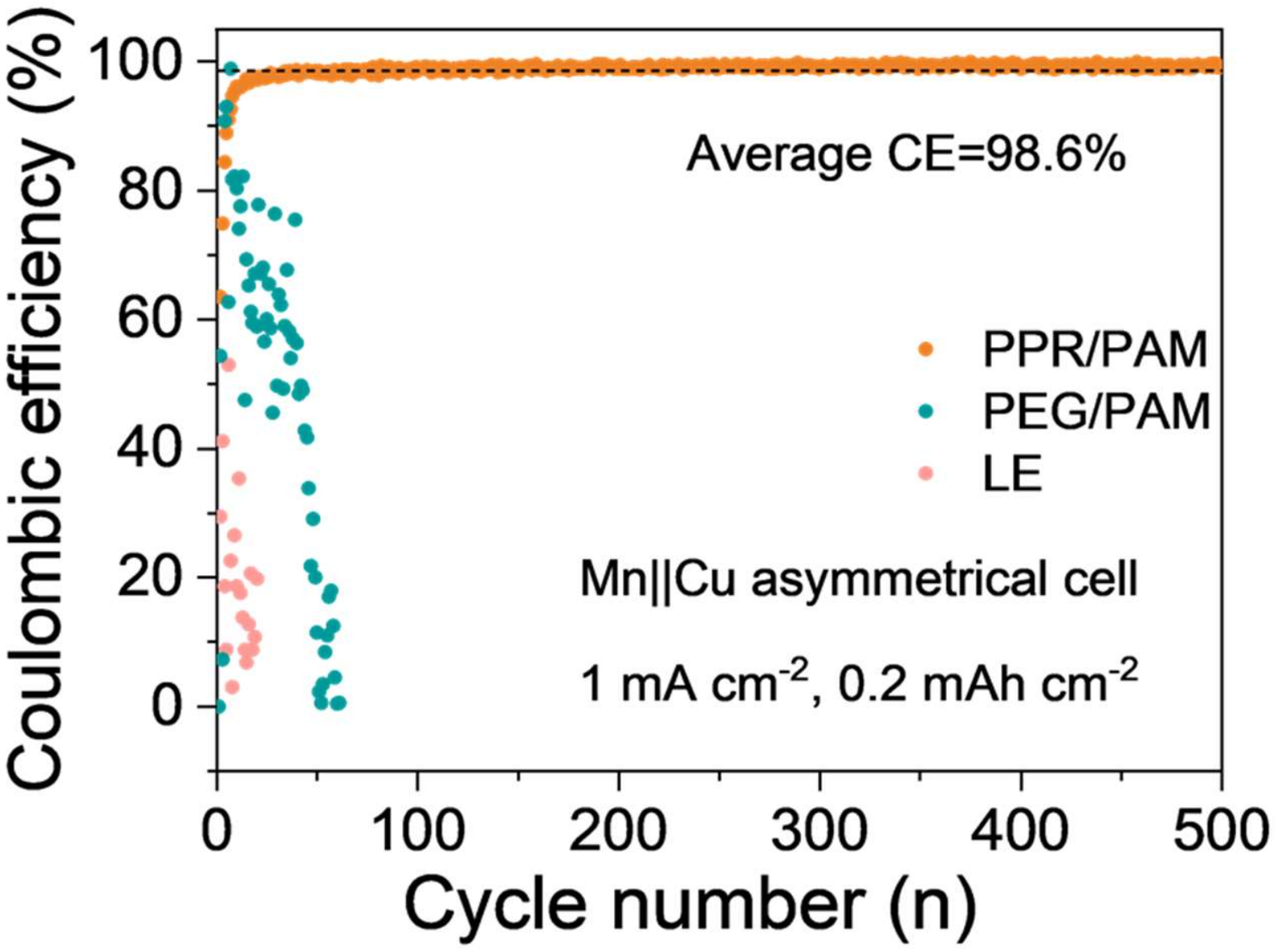

Fig. S20. Coulombic efficiencies of Mn||Cu asymmetric cells at 1 mA cm^{-2} and 0.2 mAh cm^{-2}.

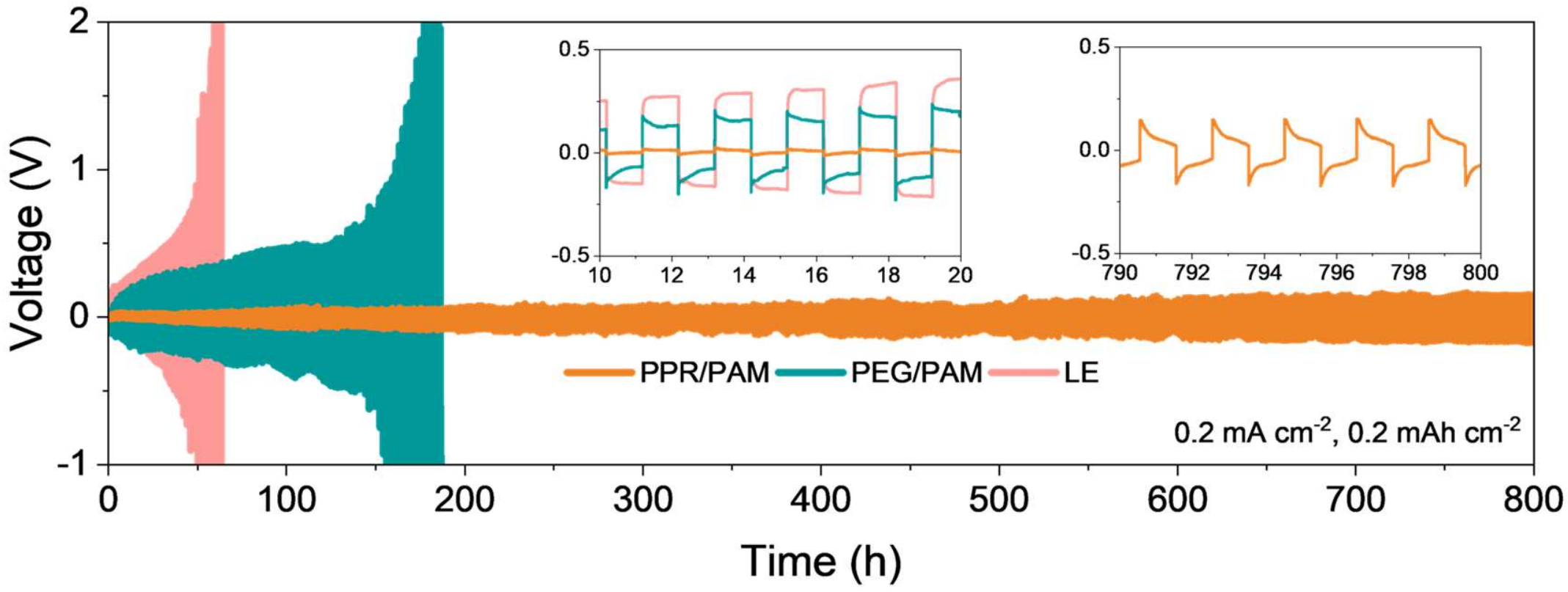

Fig. S21. Cycling performance of Mn||Mn symmetric cells in different electrolytes at 0.2 mA cm^{-2} and 0.2 mAh cm^{-2} (insets: magnified voltage profiles).

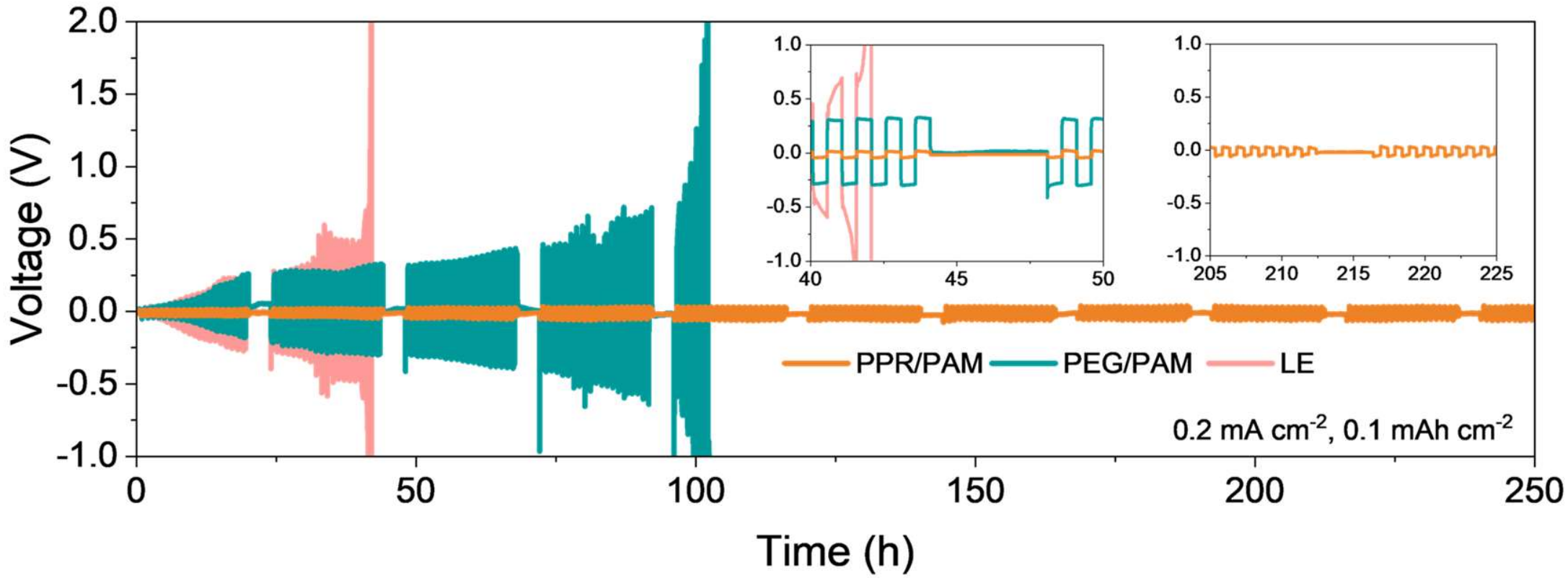

Fig. S22. Shelving recovery performance of symmetric cells (insets: magnified voltage profiles).

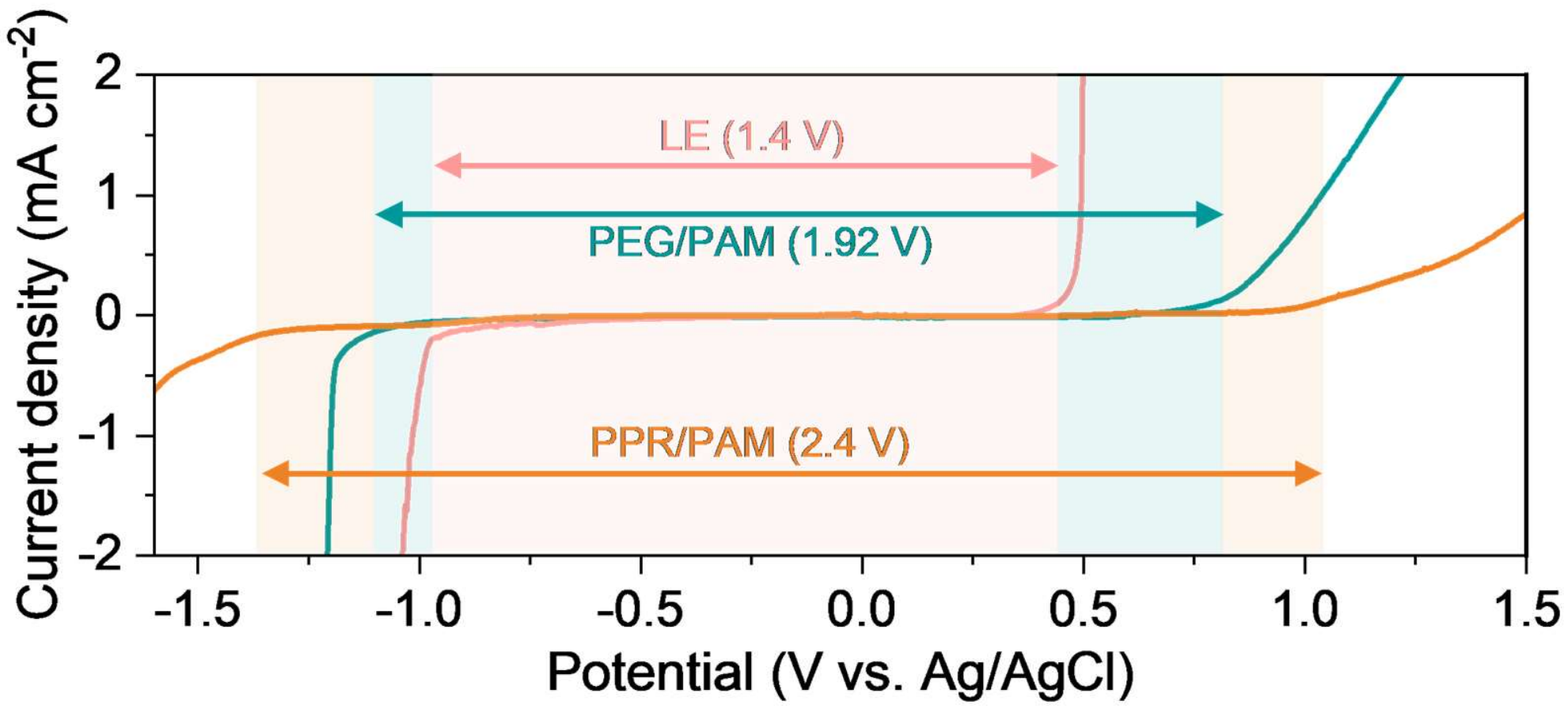

Fig. S23. Linear sweep voltammetry (LSV) curves of different electrolytes.

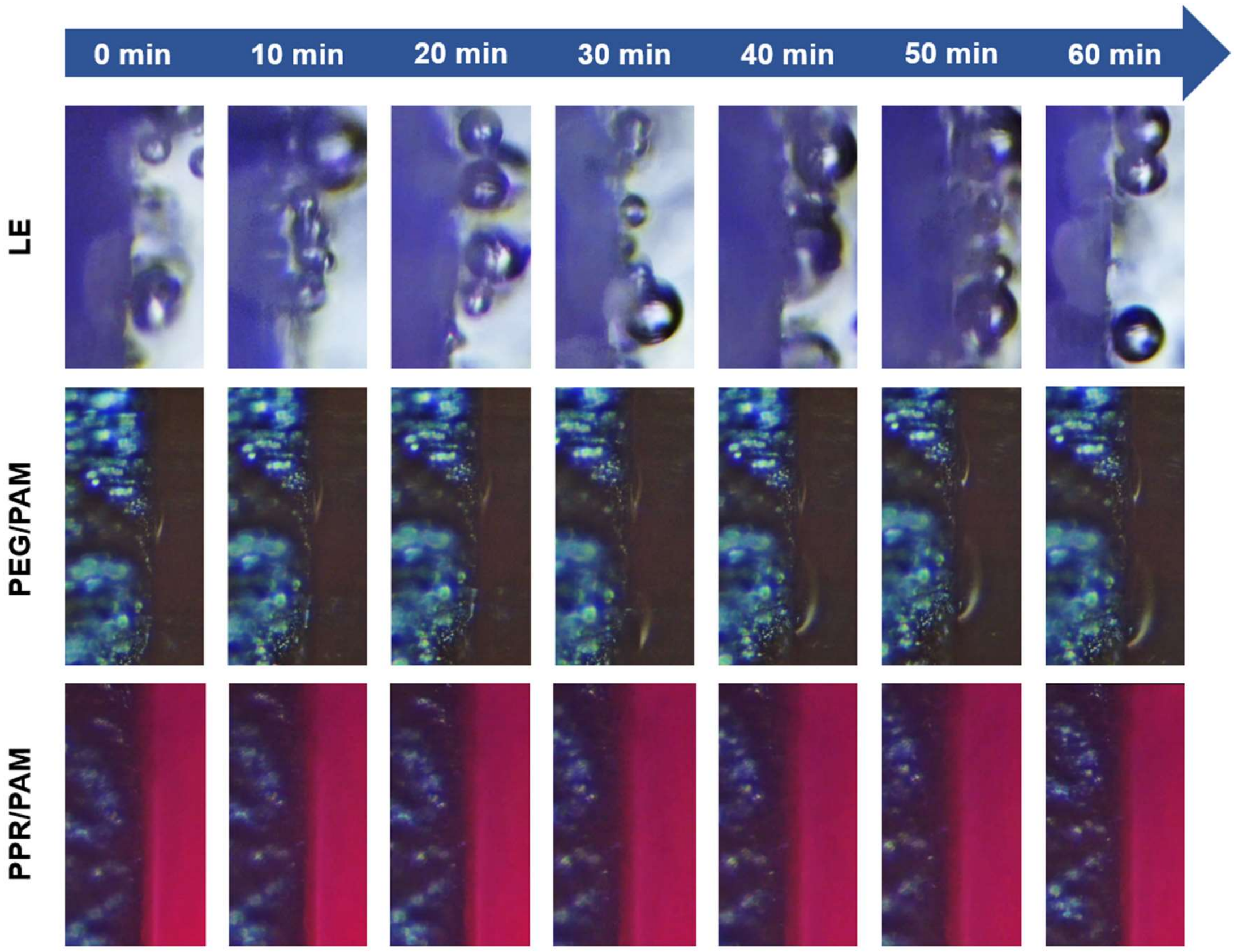

Fig. S24. Optical microscopy images of Mn electrode deposition at 0.1 mA cm^{-2} in different electrolytes.

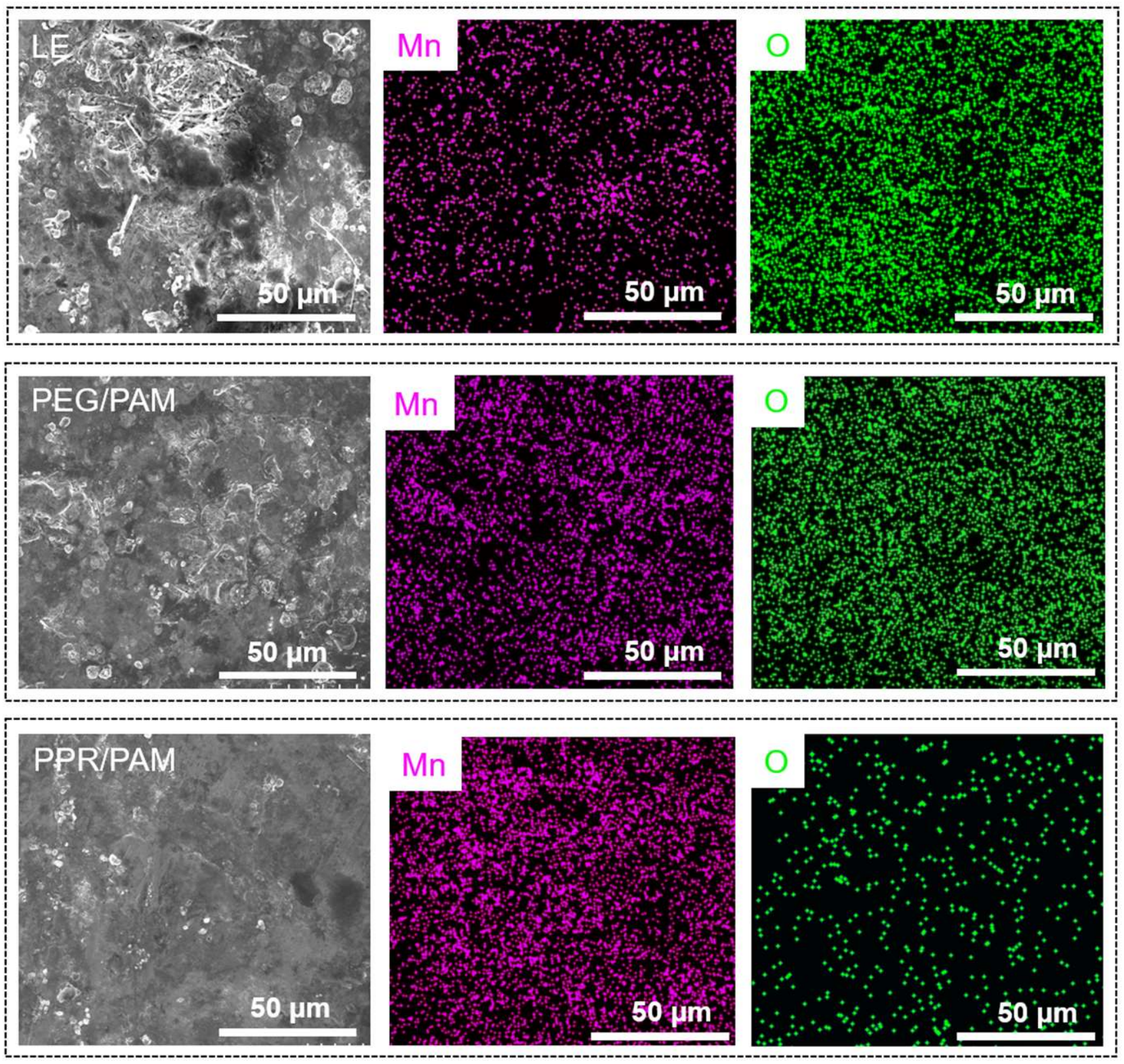

Fig. S25. Top-view SEM images and corresponding elemental mappings of Mn anodes after 100 cycles in different electrolytes.

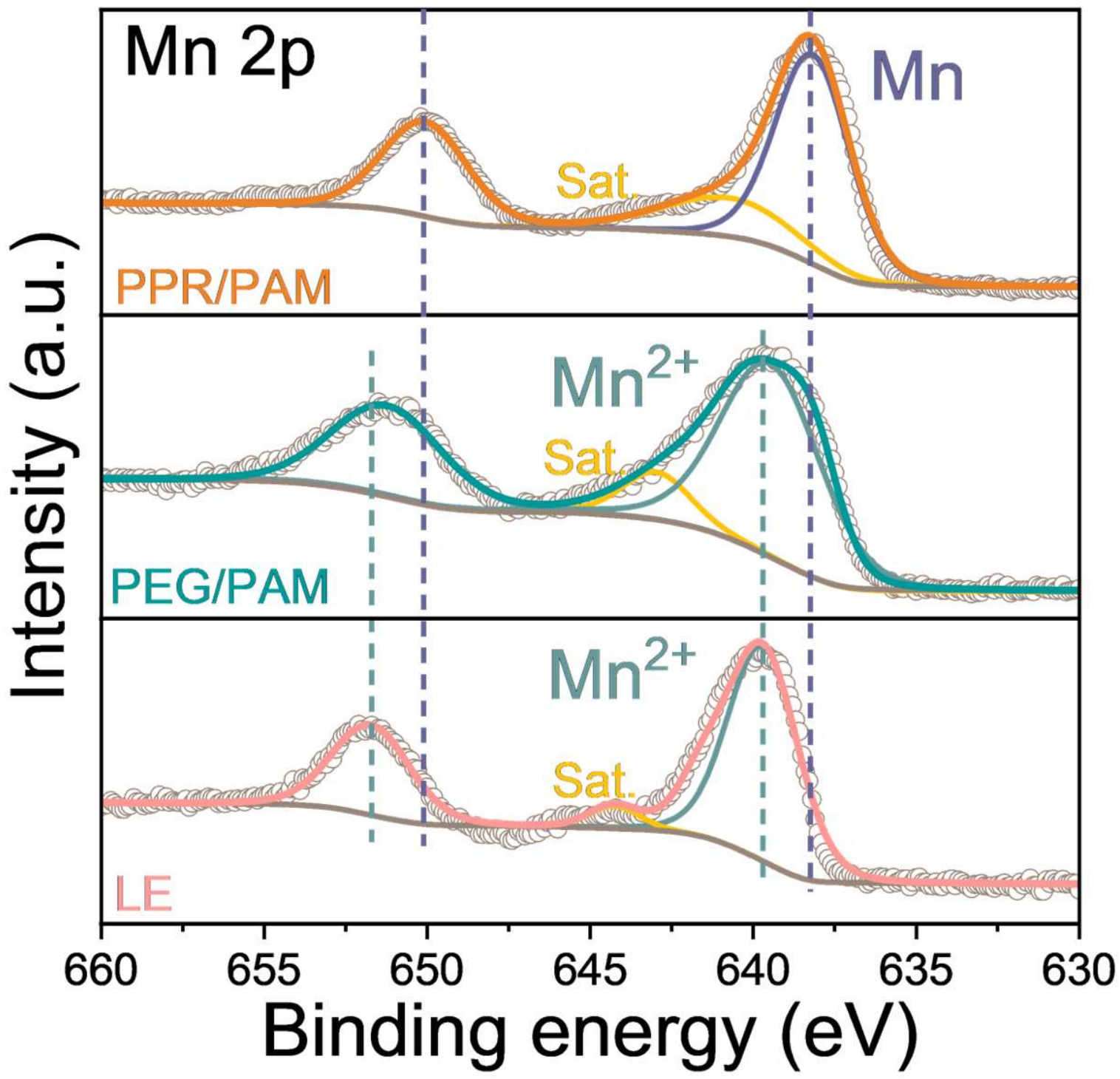

Fig. S26. XPS of Mn 2p of Mn anodes after 100 h-cycle at 0.1 mA cm^{-2} with different electrolytes.

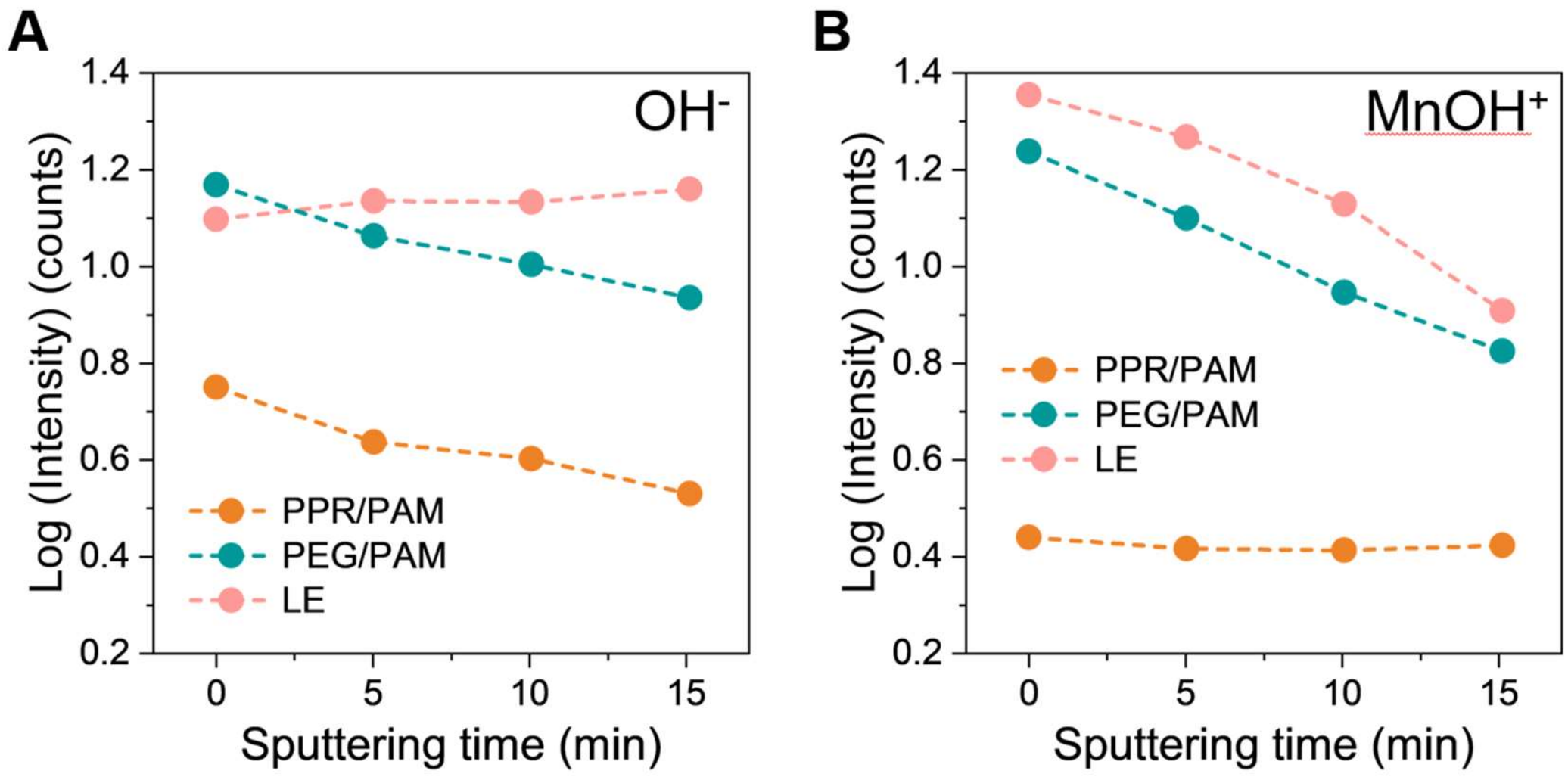

Fig. S27. Depth profiling curves of TOF-SIMS. (**A**) negative mode (**B**) positive mode.

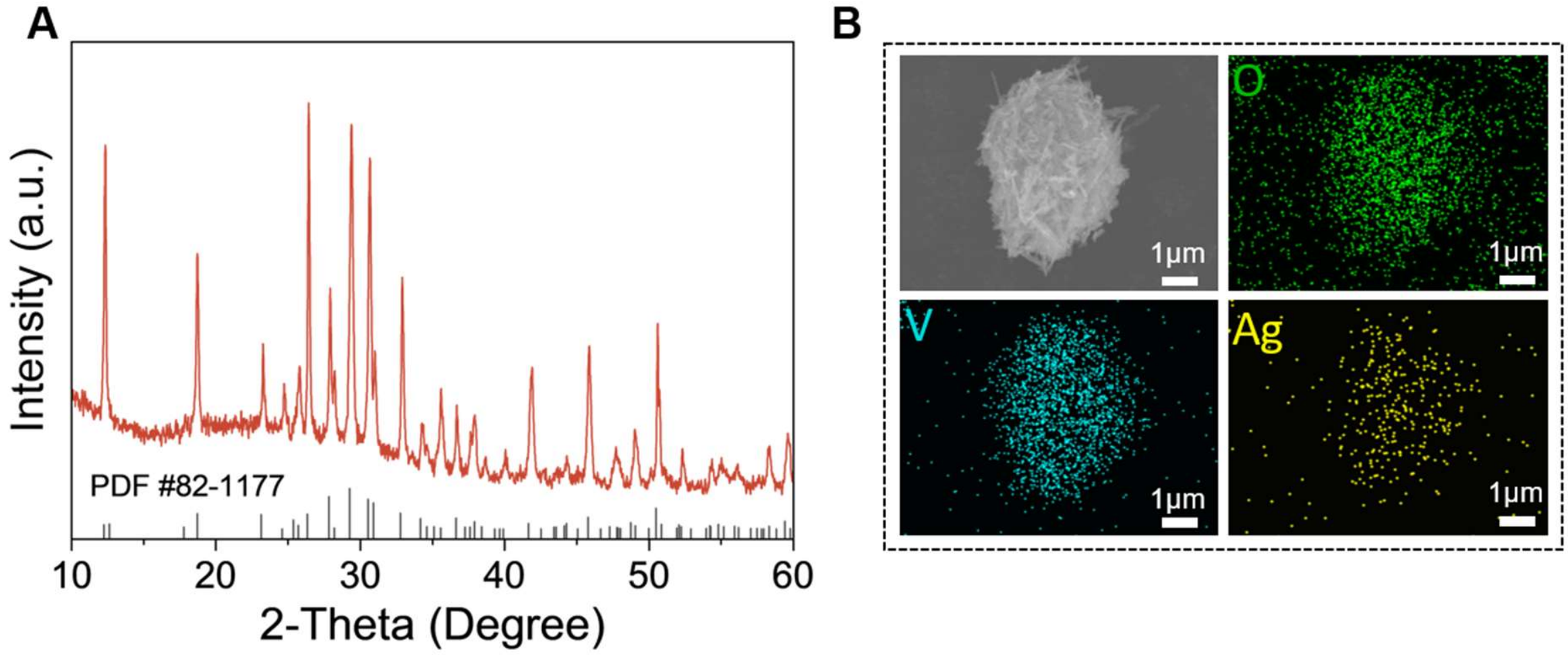

Fig. S28. Physical characterization of the AgVO cathode powder materials. (**A**) XRD pattern and (**B**) SEM with EDX mapping images.

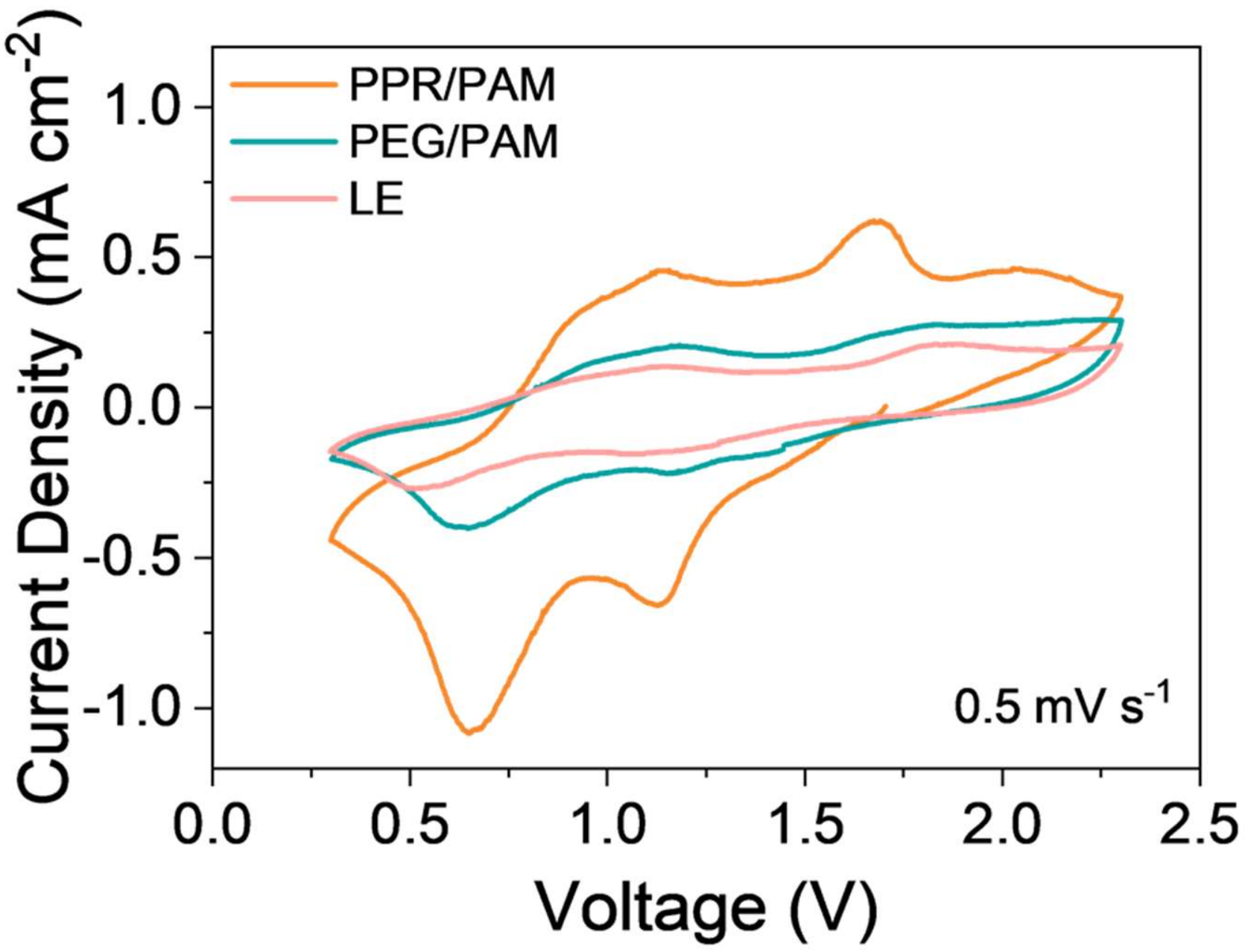

Fig. S29. Cyclic voltammetry of the Mn||AgVO full cells.

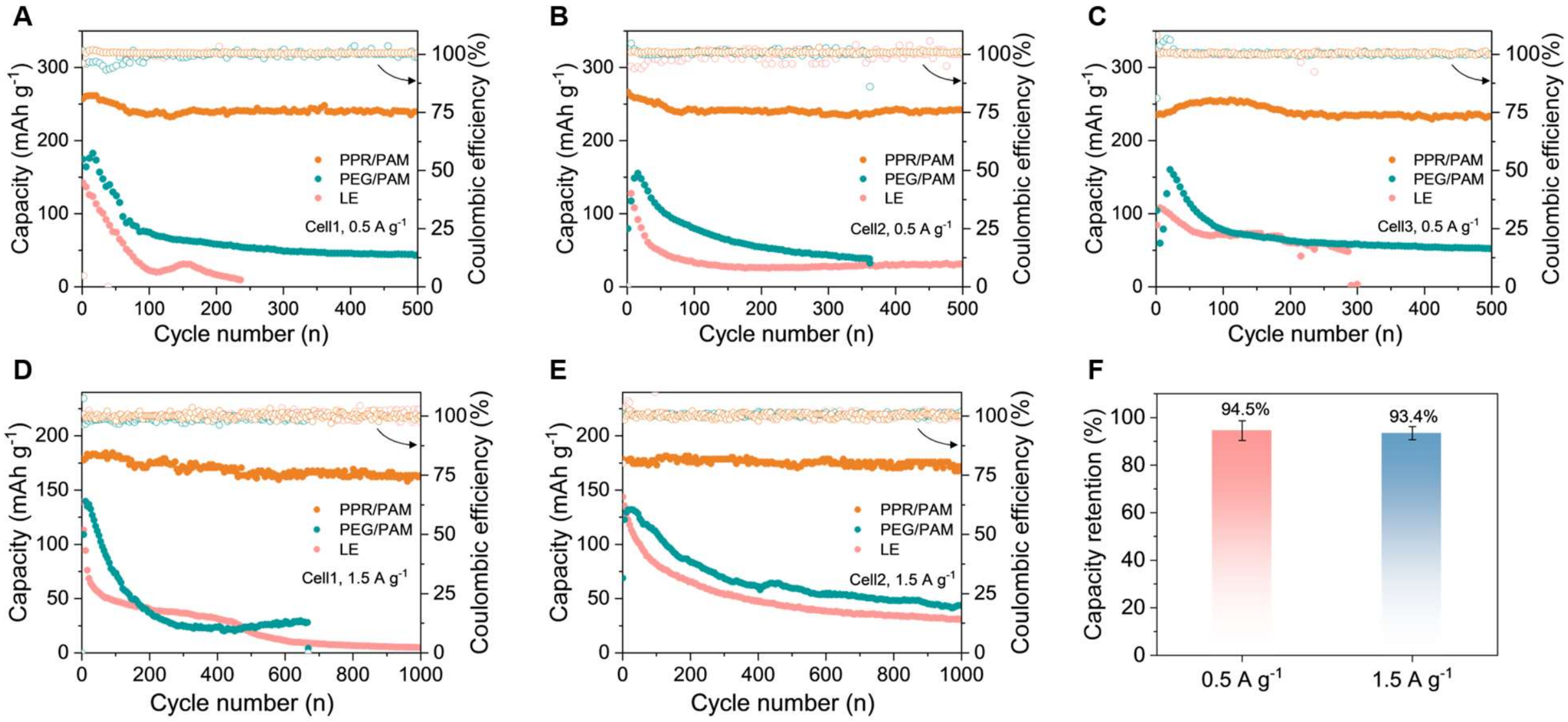

Fig. S30. Repeatability test of cycle performance of Mn||PPR/PAM||AgVO full cells. The current density of (**A-C**) 0.5 A g^{-1} and (**D, E**) 1.5 A g^{-1}. (**F**) The calculated average capacity retention at 0.5 A g^{-1}and 1.5 A g^{-1}.

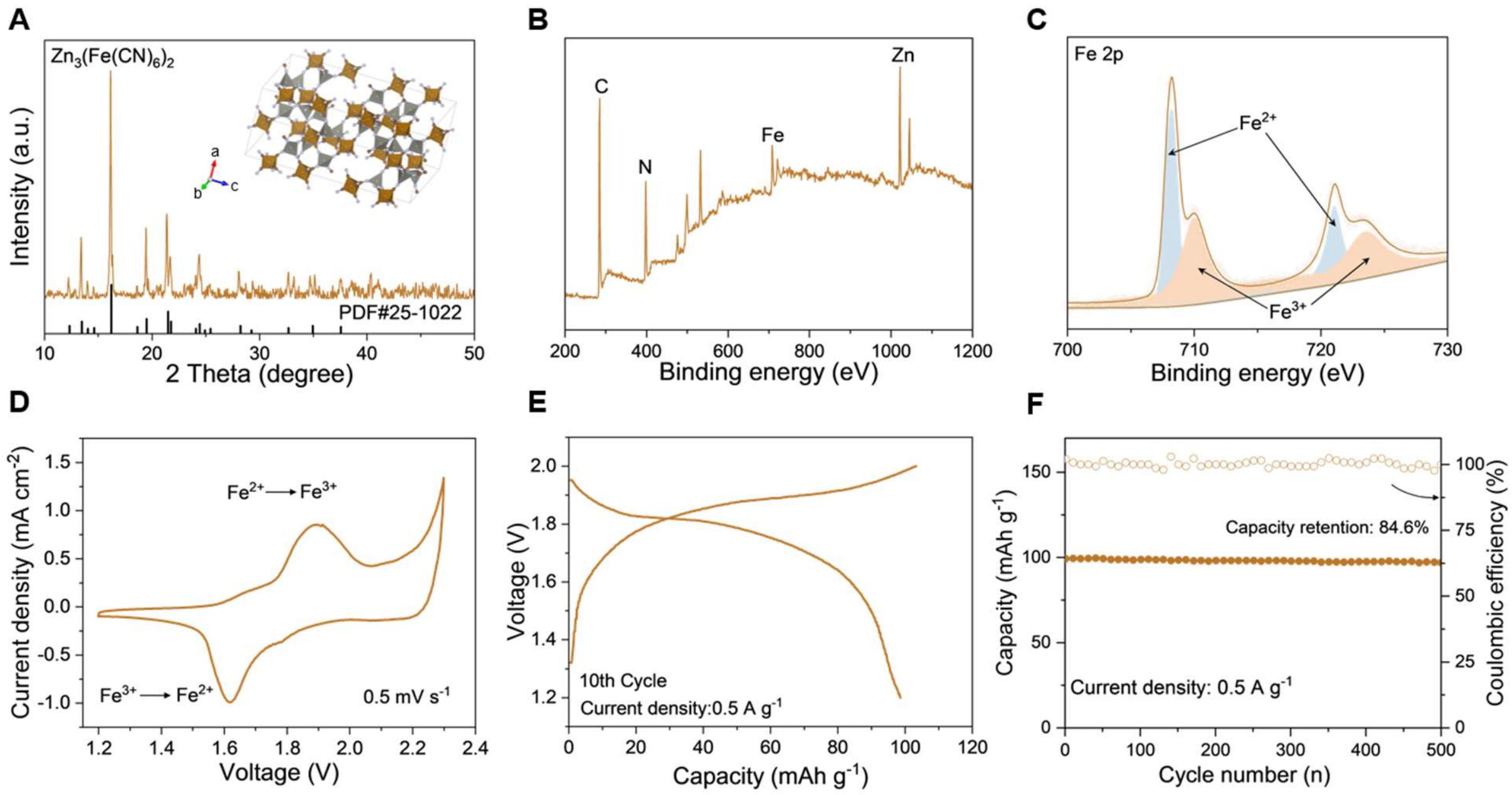

Fig. S31. Characterization and electrochemical data of Prussian blue analogue ($Zn_3(Fe(CN)_6)_2$). (**A**) XRD, (**B**) XPS survey, and (**C**) Fe 2p of the $Zn_3(Fe(CN)_6)_2$ sample. (**D**) CV curve, (**E**) GCD curve, and (**F**) cycle performance of Mn||$Zn_3(Fe(CN)_6)_2$ full cells with PPR hydrogel electrolyte.

Note: We have synthesized a Prussian blue analogue (PBA, $Zn_3(Fe(CN)_6)_2$), as a model high-voltage cathode, to further evaluate the potential of the PPR/PAM hydrogel for high-energy-density batteries. Its crystal structure was confirmed by X-ray diffraction (XRD), and X-ray photoelectron spectroscopy (XPS) revealed an iron valence state of mixed Fe (II)/Fe (III) (**Fig. S32A-C**). Full cells with the configuration Mn||$Zn_3(Fe(CN)_6)_2$, employing the PPR/PAM hydrogel, were assembled and tested. Cyclic voltammetry (CV) curves display a pair of well-defined redox peaks corresponding to the Fe^{2+}/Fe^{3+} conversion, confirming reversible high-voltage electrochemistry (**Fig. S32D**). Galvanostatic charge–discharge (GCD) profiles show a significantly higher operating plateau of 1.79 V, which is 770 mV higher than that of AgVO cathodes (**Fig. S32E**). Remarkably, the full cell delivers stable cycling performance, retaining 84.6% of its initial capacity after 500 cycles at a current density of 0.5 A g^{-1} (**Fig. S32F**). Collectively, these results convincingly demonstrate that the PPR/PAM hydrogel exhibits a wide ESW and could be successfully paired with high-voltage cathodes.

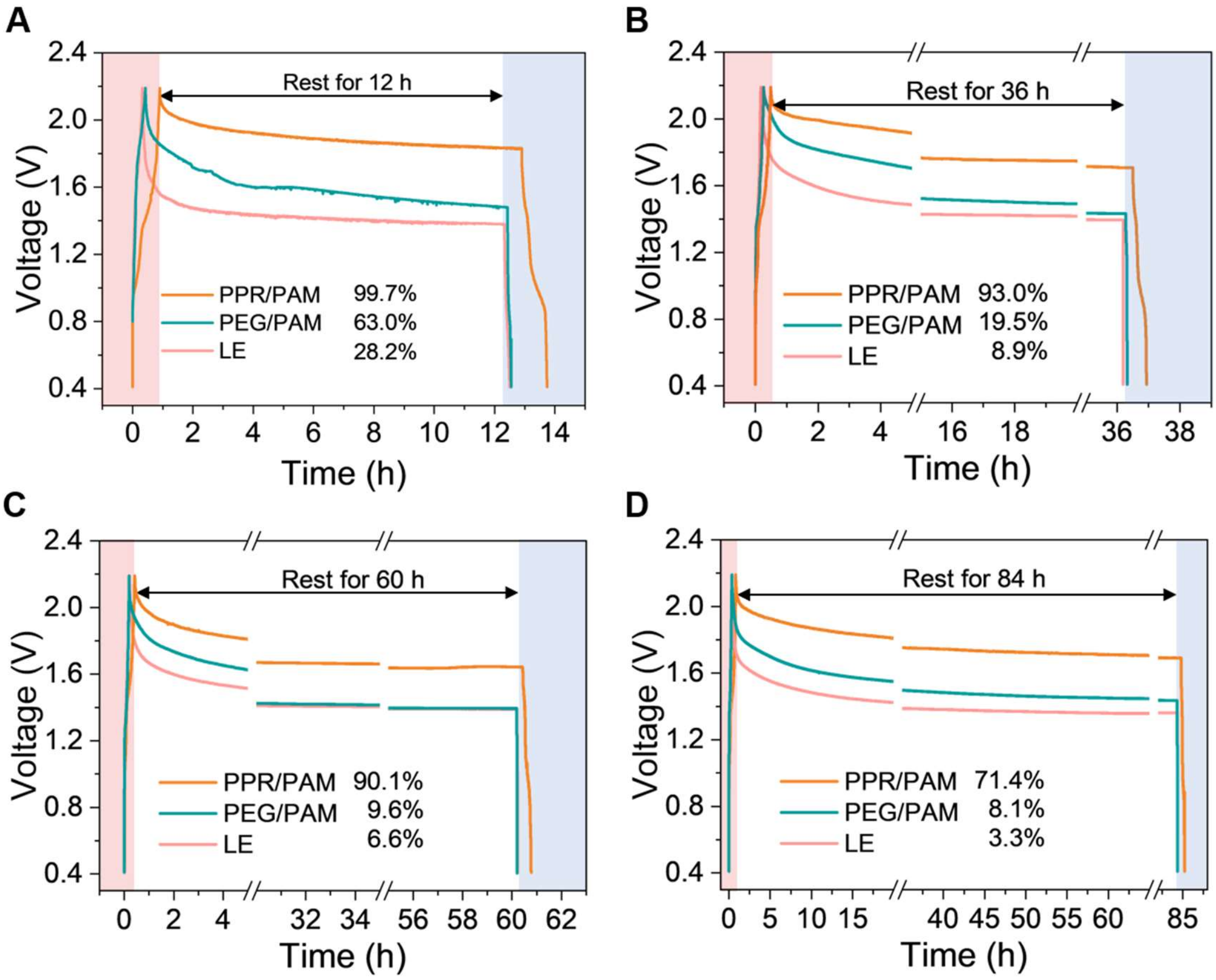

Fig. S32. Self-discharge tests of the Mn||AgVO full cells. Voltage-time curve with rest time of (**A**) 12 h, (**B**) 36 h, (**C**) 60 h, and (**D**) 84 h.

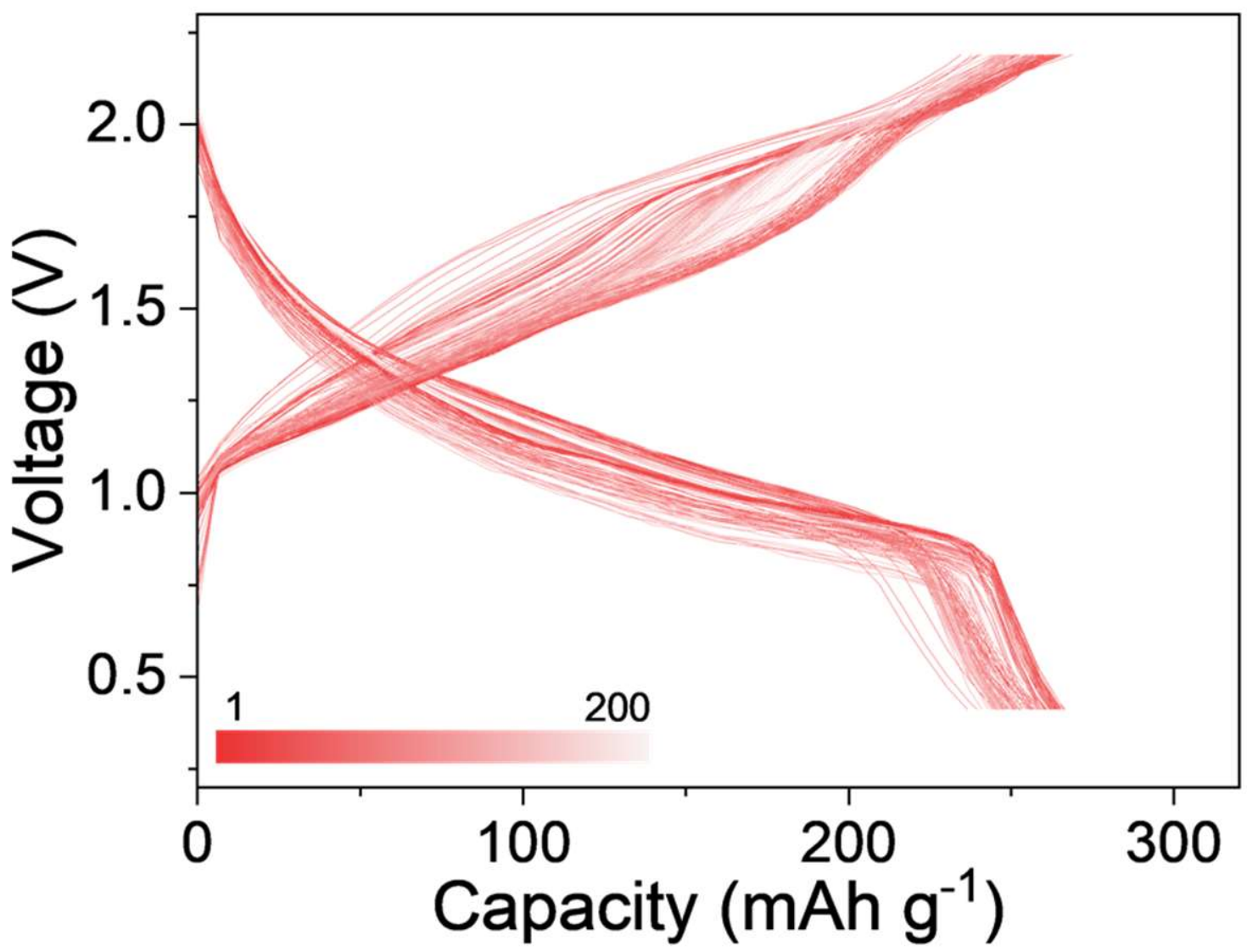

Fig. S33. The GCD potential profiles of Mn||PPR/PAM||AgVO pouch cell at a current density of 0.5 A g^{-1}.

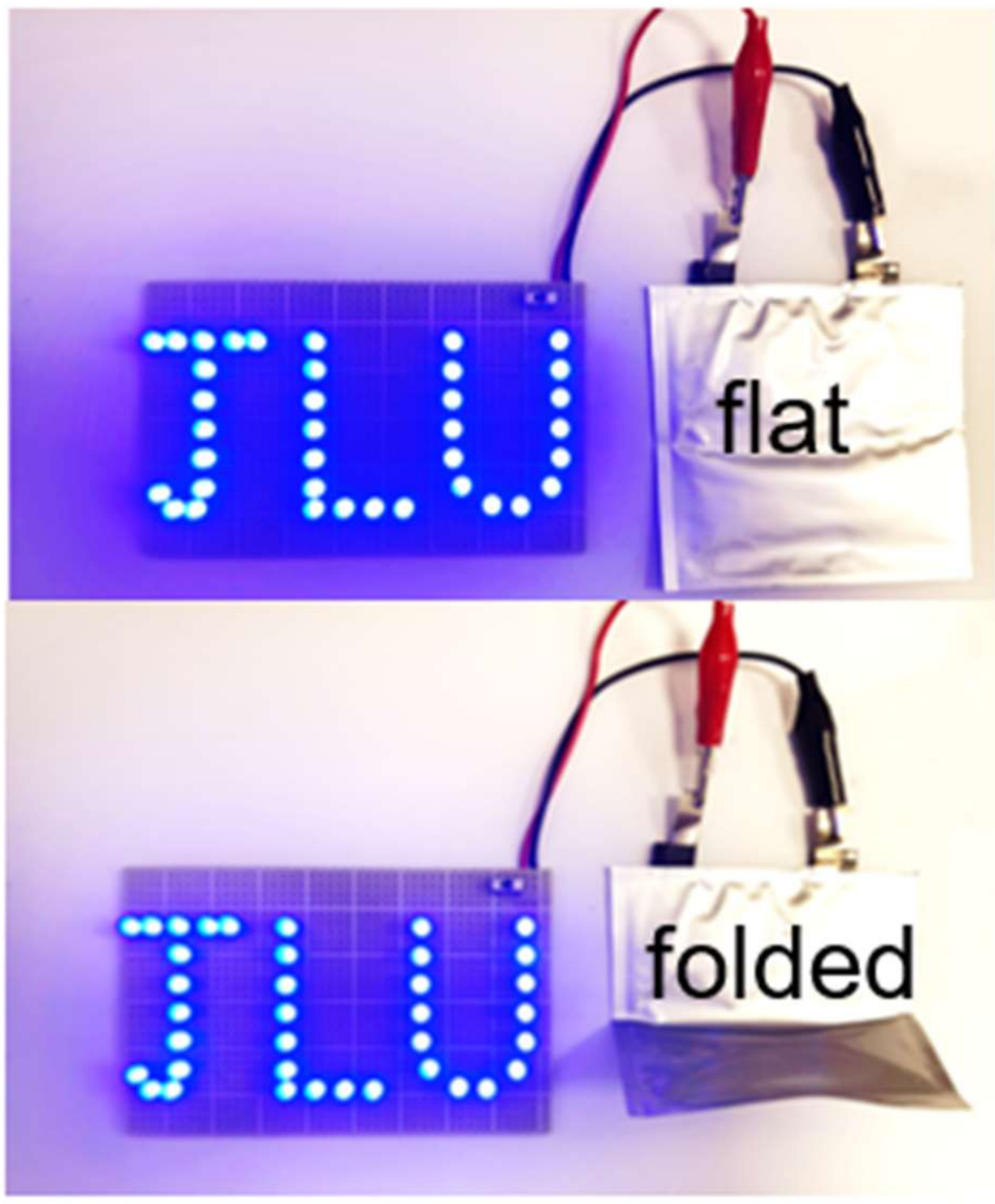

Fig. S34 Digital photos of the working flat and folded pouch cell.

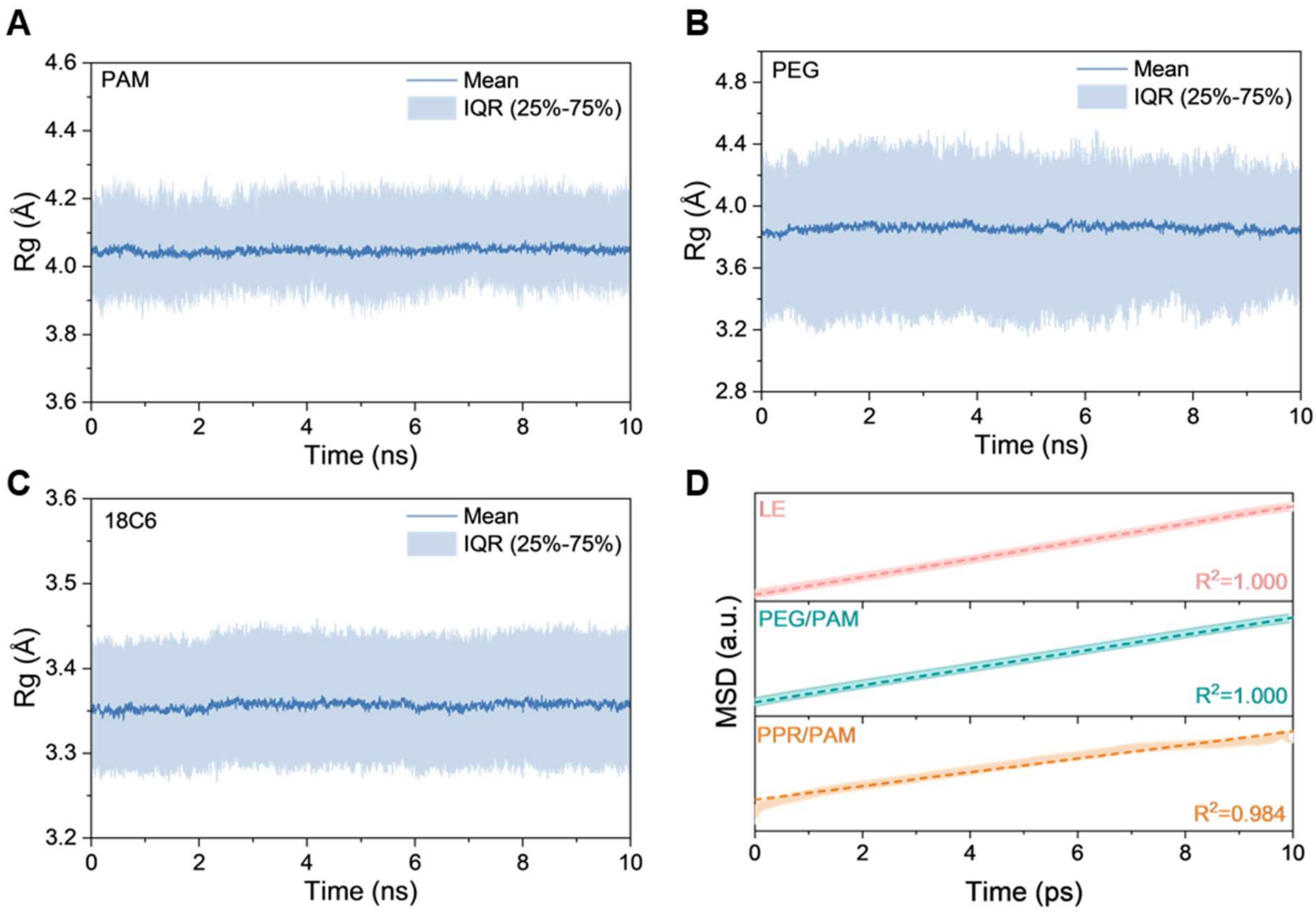

Fig. S35. Equilibrium verification of molecular dynamics system. (A-C) Time evolution of the radius of gyration (Rg) for polymer and ligand components in the PPR/PAM over 0–10 ns. Means and IQR (25–75%) are shown. **(D)** Mean square displacement (MSD) curves for the three systems.

Table S1.

Comparison of Mn anode and Zn anode.

	Zn	Mn
Mass Specific Capacity (mAh g^{-1})	820	976
Volumetric Specific Capacity (mAh cm^{-3})	5858	7250
Metal Cost (USD kg^{-1})	3.39	1.76
Element Abundance (ppm)	224	3900
Redox Potential (V *vs.* SHE)	-0.76	-1.19

Table S2.

Effect of current Mn anode stabilization strategies.

Stabilization Strategies		Cycling Time (h)	Coulomb Efficiency (%)	References
Electrolyte Engineering	SeO_2-additive $MnSO_4$ electrolyte	8.33	73%	(7)
	SA@Mn gel electrolyte	450	N/A	(8)
Artificial Interface	Sn-based interface and sucrose organic interface	300	N/A	(9)
	Mn-enriched interfacial layer	300	N/A	(10)

Table S3.

The ionic conductivity of PPR/PAM and PEG/PAM hydrogel electrolytes.

Electrolyte	Sample 1	Sample 2	Sample 3
PPR/PAM	53.7 mS cm^{-1}	52.4 mS cm^{-1}	55.2 mS cm^{-1}
PEG/PAM	33.2 mS cm^{-1}	35.3 mS cm^{-1}	31.6 mS cm^{-1}

Table S4.

The average number of H-bonds per water molecule and the average H-bond distance.

System	Avg H-bonds	Avg H-bond distance (Å)
LE	726.35	2.79
PEG/PAM	398.51	2.80
PPR/PAM (avg of 3 runs)	35.46±2.07	2.81±0.01

Table S5.

The solvation structural parameters of Mn^{2+} in three systems obtained by MD.

System	Mn–O (H_2O)			Mn–O (18C6)		
	distance (Å)	CN	g (r)	distance (Å)	CN	g (r)
LE	2.28	6.00	17.78	-	-	-
PEG/PAM	2.18	5.51	38.40	-	-	-
	2.27	1.40	29.26	2.22	4.58	95.18
PPR/PAM	2.28	1.35	29.32	2.23	4.56	92.81
	2.28	1.41	31.29	2.23	4.49	95.29

Table S6.

Comparison of Coulomb efficiency (CE) in Mn||Cu cell.

Stabilization Strategies	Current density (mA cm^{-2})	Coulombic efficiency (%)	References
PPR/PAM hydrogel electrolyte	**0.1**	**95.1 (avg)**	**This work**
	**1.0**	**98.6 (avg)**	
SeO_2-additive $MnSO_4$ electrolyte	180	73 (max)	(7)

Table S7.

Comparison of symmetric cells' cycling performance with the reported literature.

	Current Density (mA cm^{-2})	Capacity (mAh cm^{-2})	Lifespan (Hour)	Polarization Voltage (mV)	Reference
**PPR/PAM**	**0.1**	**0.1**	**1800**	**23.6**	**This Work**
	**0.2**	**0.2**	**800**	**150**	
SA@Mn	0.1	0.1	450	200	(8)
	0.2	0.2	410	600	
Mn@ISI	0.1	0.1	250	400	(9)
	0.2	0.2	150	800	
Mn@MIL	0.1	0.1	300	170	(10)
	0.2	0.2	300	220	
HM-NAE	0.1	0.1	700	116	(64)
DMF/EDA	0.1	0.1	550	600	(65)

Table S8.

Measurement of surface roughness of Mn deposited by three electrolytes.

Electrolyte types	Sa (μm)	Sz (μm)	Str	Spc (mm^{-1})	Sdr
LE	2.664	35.319	0.629	3750.646	1.166
PEG/PAM	7.033	59.004	0.820	2540.064	0.7473
PPR/PAM	0.822	17.676	0.839	1387.982	0.1692

Note: Sa (arithmetic mean height, in μm) is the average absolute deviation of height values from the mean plane. A lower Sa value indicates a smoother, more planar deposit, which homogenizes local current density and suppresses uneven nucleation. Sz (maximum height, in μm) is the sum of the highest peak and deepest valley. A small Sz confirms the absence of large isolated dendrites or pits. Sdr (developed interfacial area ratio, in %) quantifies the percentage increase of the true three-dimensional surface area relative to the projected flat area. A near-zero Sdr indicates an essentially flat interface, whereas a high Sdr reveals a rough, dendrite-covered morphology. Monitoring its change over electrochemical cycling directly assesses whether the interface remains compact. Str (texture aspect ratio, dimensionless) ranges from 0 to 1 and measures surface isotropy. A Str approaching 1 means the surface features are spatially isotropic (no preferred direction), signifying homogeneous, non-dendritic Mn deposition without directional growth. Spc (arithmetic mean peak curvature, in mm^{-1}) is the mean curvature of surface peaks. Smaller Spc values correspond to blunter, more rounded peaks, which avoid local charge concentration (tip effects) and support a stable, uniform interfacial electric field.

Table S9.

Comparison of cyclic performance with previously reported full cells.

	Current Density (A g^{-1})	Cycling number (n)	Post-cycling capacity (mAh g^{-1})	Output voltage (V)	Reference
**Mn||AgVO**	**0.5**	**500**	**250**	**1.02**	**This Work**
	**1.5**	**1000**	**160**	**0.91**	
**Mn||$Zn_3(Fe(CN)_6)_2$**	**0.5**	**500**	**100**	**1.79**	
Mn||AgVO	0.5	200	200	1.00	(8)
	1.5	410	150	0.90	
Mn@ISI||V_2O_5	0.5	60	180	~1.00	(9)
	1.5	200	100	N/A	
Mn@MIL||V_2O_5	0.5	110	300	N/A	(10)
	1.0	200	200	N/A	
Mn||Mo_6S_8	0.2	600	91.4	~0.50	(64)
InN@Mn||PTO	0.1	400	84.3	~0.80	(66)
